# Regulation of RNase E during the UV stress response in the cyanobacterium *Synechocystis* sp. PCC 6803

**DOI:** 10.1002/mlf2.12056

**Published:** 2023-02-15

**Authors:** Satoru Watanabe, Damir Stazic, Jens Georg, Shota Ohtake, Yutaka Sakamaki, Megumi Numakura, Munehiko Asayama, Taku Chibazakura, Annegret Wilde, Claudia Steglich, Wolfgang R. Hess

**Affiliations:** ^1^ Faculty of Biology, Genetics and Experimental Bioinformatics University of Freiburg Freiburg Germany; ^2^ Department of Bioscience Tokyo University of Agriculture Setagaya‐ku Tokyo Japan; ^3^ School of Agriculture, Molecular Genetics Ibaraki University Ibaraki Japan; ^4^ Faculty of Biology, Molecular Genetics University of Freiburg Freiburg Germany; ^5^ Present address: Nexxiot Prime Tower (Hardstrasse 201) Zurich Switzerland

**Keywords:** cyanobacteria, protein turnover, ribonuclease, stress response

## Abstract

Endoribonucleases govern the maturation and degradation of RNA and are indispensable in the posttranscriptional regulation of gene expression. A key endoribonuclease in Gram‐negative bacteria is RNase E. To ensure an appropriate supply of RNase E, some bacteria, such as *Escherichia coli*, feedback‐regulate RNase E expression via the *rne* 5′‐untranslated region (5′ UTR) in *cis*. However, the mechanisms involved in the control of RNase E in other bacteria largely remain unknown. Cyanobacteria rely on solar light as an energy source for photosynthesis, despite the inherent ultraviolet (UV) irradiation. In this study, we first investigated globally the changes in gene expression in the cyanobacterium *Synechocystis* sp. PCC 6803 after a brief exposure to UV. Among the 407 responding genes 2 h after UV exposure was a prominent upregulation of *rne* mRNA level. Moreover, the enzymatic activity of RNase E rapidly increased as well, although the protein stability decreased. This unique response was underpinned by the increased accumulation of full‐length *rne* mRNA caused by the stabilization of its 5′ UTR and suppression of premature transcriptional termination, but not by an increased transcription rate. Mapping of RNA 3′ ends and in vitro cleavage assays revealed that RNase E cleaves within a stretch of six consecutive uridine residues within the *rne* 5′ UTR, indicating autoregulation. These observations suggest that RNase E in cyanobacteria contributes to reshaping the transcriptome during the UV stress response and that its required activity level is secured at the RNA level despite the enhanced turnover of the protein.

## Impact statement

Cyanobacteria use solar light for oxygenic photosynthesis and therefore are inevitably exposed to UV radiation. Our transcriptomic analysis revealed hundreds of genes with changed expression 2 h after exposure to UV‐C. Several of these genes encode proteins involved in RNA metabolism, with RNase E as a prominent example. The results indicate for the first time that UV‐induced stress impacts the ability of cyanobacterial RNase E to autoregulate its expression, mRNA level, and activity.

## INTRODUCTION

mRNA degradation plays a key and universal role in the posttranscriptional control of gene expression. In both prokaryotic and eukaryotic organisms, mRNA lifetime can vary by up to two orders of magnitude, with proportionate effects on protein production^1^. In *Escherichia coli*, mRNA decay mechanisms involving the sequential action of endonucleases and 3′ exonucleases have been well studied^2^, ^3^. The endonuclease that is most important for mRNA turnover in *E. coli* is endoribonuclease (RNase) E. In addition to its function in the degradation of most mRNAs, RNase E also participates in ribosomal RNA (rRNA) and transfer RNA maturation^4^. *E. coli* RNase E cuts RNA within single‐stranded regions that are AU‐rich, although the presence of a guanosine residue two nucleotides upstream of the cleavage site increases reactivity^5^, ^6^, ^7^, ^8^. The core motif recognized by RNase E of *Salmonella typhimurium*, a close relative of *E. coli*, has been specified as “RN ↓ WUU”; the enzyme shows a marked preference for uridine at position +2 after the cleavage site (indicated by ↓)^9^. RNase E is also the key enzyme in the interactions between bacterial regulatory small RNAs (sRNAs) and their targets, to which it can be recruited upon sRNA binding^10^ or excluded from accessing possible cleavage sites^11^, ^12^. In view of its many crucial biological functions, it is not surprising that *rne*, the gene encoding RNase E, is essential in *E. coli* and that imbalanced production of RNase E can impede cell growth^13^, ^14^, ^15^. To ensure a steady supply of RNase E, *E. coli* and related bacteria have evolved a homeostatic mechanism for tightly regulating its synthesis in which the level and rate of decay of *rne* mRNA are modulated in response to changes in cellular RNase E activity^16^. The feedback regulation of RNase E is mediated by the *rne* 5′ UTR in *cis* ^16^, ^17^. Compared to the 5′ UTRs of other genes, the 5′ UTR of *E. coli rne* is very long at 361 nucleotides^18^. Through its cleavage by RNase E and its expediting of cleavage elsewhere within the *rne* transcript, this long 5′ UTR is critically involved in the control of RNase E synthesis^19^.

Cyanobacteria are the only bacteria that perform oxygenic photosynthesis similar to the photosynthesis that occurs in plant and algal chloroplasts. Cyanobacterial RNase E proteins are smaller than their homologs in most other bacteria: the RNase E of the cyanobacterium *Synechocystis* sp. PCC 6803 (hereafter *Synechocystis* 6803) consists of 674 amino acid residues, whereas RNase E of *E. coli* and *Pseudomonas* spp. contain more than 1000 residues. This more compact form of RNase E is highly conserved among cyanobacteria and occurs in plant and algae, where chloroplast import is mediated via an additional N‐terminal targeting sequence^20^. A detailed introduction into the function of RNase E and RNA degradation in cyanobacteria and model bacteria has recently been published^21^. In the cyanobacterium *Synechocystis* 6803, complete genetic disruption of the *rne* gene (gene *slr1129*) failed to segregate into a homozygous mutant line^22^, ^23^, and partial disruption of RNase E led to severe growth inhibition and affected the expression of a large number of genes^23^, indicating that RNase E is essential. Comprehensive transcriptome analyses revealed the transcriptional start sites (TSSs) of *rne* and indicated that, compared to *E. coli* (361 nt), even longer 5′ UTRs (458–622 nt) are typically associated with the *rne* gene in several different cyanobacteria, such as *Synechocystis* sp., *Anabaena* sp., and *Synechococcus elongatus* ^24^, ^25^, ^26^, ^27^ (Figure S1A). However, the molecular details of a possible existing regulation linked to these long 5′ UTRs have remained unknown.

RNase E in *Synechocystis* 6803 participates in the posttranscriptional regulation of *psbA2*, which encodes the photosystem (PS) II reaction center D1 protein. In the dark, when *psbA2* expression is not required, RNase E cleaves at two tightly spaced sites, the AU box and within the ribosome binding site, both of which are located in the 5′ UTR of the *psbA2* transcript^28^, ^29^. However, these sites are not cleaved when the cells are cultivated in the light and *psbA2* expression is high^28^, ^29^. PsbA2R and PsbA3R, two *cis*‐encoded antisense RNAs (asRNAs), are involved in the stabilization of *psbA2* and *psbA3* transcripts in the light, and this protective effect has physiological relevance^30^. Another function rr55threlevant to the proper functioning of the photosynthetic apparatus is the recruitment of RNase E upon binding of the sRNA PsrR1 to a cleavage site located four nucleotides downstream of the start codon within the *psaLI* dicistronic mRNA encoding two PS I proteins^31^.

RNase E has also been shown to be involved in the processing of polycistronic transcripts. In *Synechocystis* 6803, the DEAD‐box RNA helicase CrhR responds to cold stress^32^, ^33^. The *crhR* gene forms an operon with *rimO*, which encodes a methylthiotransferase. In vitro cleavage experiments suggested that RNase E cleaves the polycistronic *rimO‐crhR* transcript and that it is required for the autoregulation of CrhR expression^34^. Another critical role of RNase E in *Synechocystis* 6803 is involved in the clustered regularly interspaced short palindromic repeat (CRISPR)‐Cas defense mechanism, where it is involved in the maturation of CRISPR‐derived RNAs (crRNAs)^35^.

RNase E thus appears to play a pivotal role in cyanobacteria. Indeed, the recent mapping of RNase E‐dependent cleavage sites in *Synechocystis* 6803 after transient inactivation of RNase E by temperature shift (TIER‐seq) yielded 1472 such sites^36^. The dominant cleavage signature was found to consist of an adenine at the −3 position and a uridine at the +2 position within a single‐stranded segment of the RNA^36^.

As an energy source for their photosynthetic lifestyle, cyanobacteria rely on solar energy. Under natural conditions, light intensity varies frequently and substantially, as does the inherent fraction of ultraviolet (UV) light. Therefore, cyanobacteria must use specific mechanisms to cope with UV light‐induced damage to biomolecules. Since nucleic acid molecules (not only DNA but also RNA) are primary targets of UV radiation^37^ and damaged RNA may perturb cellular gene expression^38^, it is reasonable that RNase E becomes activated after UV treatment. In *Synechocystis* 6803, *rne* transcript levels increased approximately two to three folds after treatment with UV‐B as well as UV‐C^39^, ^40^, but this also occurred following sulfur starvation^41^ or redox stress^42^. However, neither the functional relevance of the enhanced expression of *rne* under these conditions nor the mechanisms underlying it have been elucidated.

Here, we demonstrate that the UV stress response in *Synechocystis* 6803 involves dynamic changes in the transcriptome and triggers feedback regulation of RNase E. Following brief UV irradiation, full‐length forms of *rne* mRNA started to accumulate; this was caused by selective stabilization of its 5′ UTR and suppressed premature termination of *rne* transcription, while an increased transcription rate was not involved. In parallel, the activity of RNase E increased while the amount of RNase E protein remained constant, although RNase E protein stability decreased. Mapping of RNA 3′ ends and in vitro cleavage assays indicated that *Synechocystis* 6803 RNase E cleaves close to and within a U‐rich region in the 5′ UTR of *rne* mRNA. Our findings suggest that RNase E is involved in reshaping the transcriptome during the UV stress response in cyanobacteria and that its required activity level is ensured despite enhanced turnover of the protein. The underlying mechanism involves feedback regulation acting on a U‐rich element within the *rne* 5′ UTR.

## RESULTS

### Microarray analysis and increased *rne* transcript levels after UV irradiation

Clear changes in gene expression were previously observed in *Synechocystis* sp. PCC 6803 following exposure to UV‐B^40^ as well as UV‐C^39^. In both studies, the *rne* gene has been reported to be induced in *Synechocystis* 6803 by UV stress^39^, ^40^. In this study we chose to apply UV‐C (254 nm) because at this wavelength, direct effects on nucleic acids including RNA can be expected and we aimed to characterize the role of RNase E in the response to this severe stress condition.

We first conducted a UV‐C irradiation assay by exposing cells from exponentially growing cultures to 400–16,000 J/m^2^ UV irradiation. Colony formation assays indicated that after irradiation at 400 J/m^2^, the survival rate was 80% (Figure S2); thus, this intensity of irradiation was used in all subsequent experiments.

**Figure 1:**
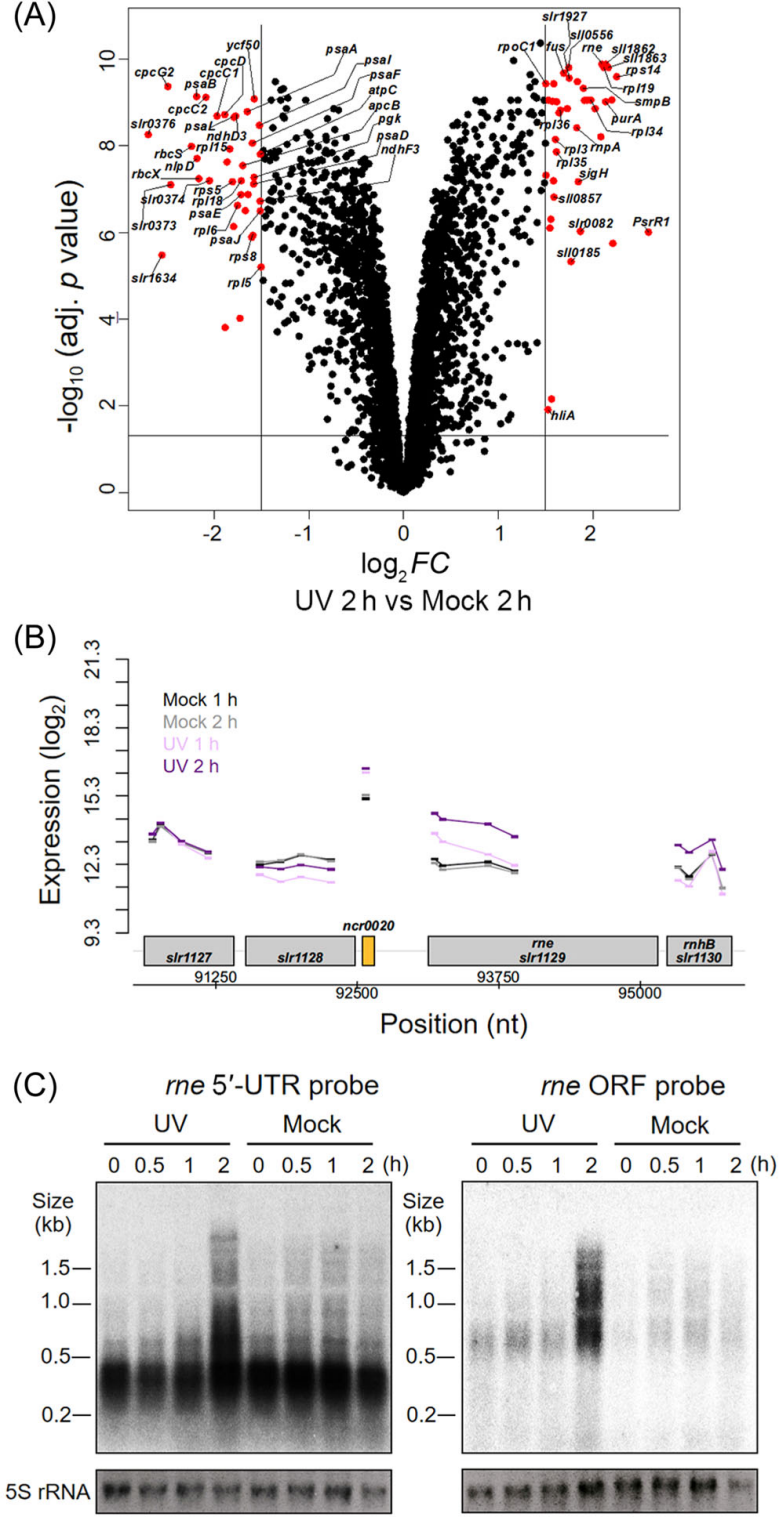
Transcriptomic response to UV treatment and increased accumulation of the *rne* transcript. (A) Volcano plot: log‐transformed fold changes (FCs) between samples taken 2 h after UV irradiation and after mock treatment (*x*‐axis, difference in log_2_ expression values; *y*‐axis, −log_10_ (adjusted *p* value)). The lines indicate the adjusted *p* value threshold of 0.05 and the *FC* thresholds of 1 and −1. The entire data set is shown in the genome‐wide expression plot (Data S2), and numerical values are presented in Data S1. (B) Detailed view of the *rne* locus with array probes indicated by horizontal bars connected by colored lines. All genes are located in the (+) strand direction. The *rne* gene, which is transcribed together with the *rnhB* gene encoding RNase H, has a long 5′ UTR from which separate shorter transcripts can also accumulate, annotated as *ncr0020*. The signal intensities are given as log_2_ values. (C) Northern blot analysis of *rne* expression after UV treatment using single‐stranded RNA probes that hybridize either to the *rne* 5′ UTR or to the coding region (ORF) (Figure S4A and Table S2). 5S rRNA is shown as a control. The High Range RNA Ladder (Thermo‐Fisher) was used as the size standard. One representative blot out of three is shown. rRNA, ribosomal RNA; UTR, untranslated region; UV, ultraviolet.

A microarray analysis in which cells were subjected to mock condition without UV treatment or UV stress treatments revealed dynamic transcriptome changes during the UV stress response. One hour after UV irradiation, we observed 275 upregulated and 306 downregulated genes, and at 2 h after the initiation of UV stress, we observed 189 upregulated and 218 downregulated genes (log_2_ *FC* ≥ │1│, adj. *p* ≤ 0.01; Supporting information: Data S1). Because the microarray also contained probes that detect UTRs and noncoding transcripts, the term “gene” here includes not only protein‐coding genes but also sRNAs and separate UTRs. A genome‐wide graphical overview of probe localization and signal intensities is shown in Supporting information: Data S2. A volcano plot indicating log‐transformed fold changes (FCs) at 2 h after UV treatment (compared to cells that received mock treatment) is shown in Figure 1A. Several genes classified in the Cyanobase^43^, ^44^ GO categories “translation” and “photosynthesis and respiration”, including gene clusters encoding ribosomal proteins, ATPase subunits, and RubisCo subunits, were considerably downregulated 1 h after UV irradiation (Figure S3A–C, Data S1). In contrast, *slr1639*, which encodes SmpB, a protein that binds to tmRNA (*ssrA* RNA) and works in concert with it to rescue stalled ribosomes^45^, was upregulated, as were some specific ribosomal genes (Figure S3D, Data S1). These results suggest that translation arrest and reconstruction of the ribosome occurred during this period. Several sRNAs were also upregulated at these time points. Among them, PsrR1, a negative posttranscriptional regulator of multiple PSI genes in response to high light stress^31^, was induced, and also HLIP genes (*hliA*: *ssl2542*; *hliB*: *ssr2595*; *hliC*: *ssl1633*) (Figure S3E–H), the products of which quench absorbed light energy and aid chlorophyll biosynthesis and PS II assembly^46^. In addition, the sRNA *ncl0380*, which corresponds to the 5′ UTR of *sll1799* encoding ribosomal protein L3, was upregulated at both 1 and 2 h after UV treatment, while the downstream‐located ribosomal gene cluster was strongly downregulated at 1 h after UV treatment and then showed a gradient of differential partial recovery over time (Figure S3A); this recovery was most pronounced for the first genes in the cluster and strongly decreased toward the end of the operon. Interestingly, we did not observe differences in the transcript levels of *lexA*, consistent with previous observations that the *Synechocystis lexA* gene is not induced by DNA damage^47^. Regarding the regulation of genes that encode ribonucleases, we observed the prominent upregulation of *rne* 2 h after UV irradiation (Figure 1B), consistent with a previous report^40^. Likewise, the *rnj* gene (*slr0551*) encoding RNase J (Figure S3I) was also upregulated at this time point. RNase E and RNase J are key enzymes in RNA metabolism. Therefore, their enhanced transcript accumulation likely indicates their involvement in transcriptome remodeling following UV‐induced damage. We also noted a unique UV stress response of the *rimO‐crhR* operon, one of the targets of RNase E^34^. The *rimO* transcript level was significantly upregulated after UV treatment, whereas *crhR* mRNA levels responded in an inverse fashion by transiently decreasing 1 h after UV treatment (Figure S3J). This is consistent with a previous observation of posttranscriptional operon discoordination in the UTR between *rimO* and *crhR* ^34^.

We next investigated the UV stress response of the *rne* gene in more detail. Northern blot analyses using probes specific for the *rne* 5′ UTR or the coding sequence 5′ portion (Figure S4A) revealed that transcripts in the 200–500 nt range originating from the 5′ UTR were abundant under all tested conditions (Figure 1C, left). In contrast, the *rne* full‐length transcript became detectable 2 h after UV treatment (Figure 1C, right, UV), whereas the *rne* transcript steady‐state level remained low in the mock condition without UV treatment (Figure 1C, right, mock). The largest distinct mRNA, with a length of ~3.2 kb, was seen 2 h after UV treatment (Figure 1C); this mRNA originates from the dicistronic transcriptional unit that consists of *rne* and the downstream‐located *rnh* gene encoding RNase H^48^. Consistent with previous reports^39^, these results indicate that *rne* expression is upregulated after UV‐C irradiation and that the *rne* 5′ UTR accumulates as an abundant and separate transcript, consistent with previous transcriptome data in *Synechocystis* 6803 (Figures 1B and S1B)^26^ and *Synechocystis* sp. PCC 6714 (Figure S1B)^49^, a closely related strain^48^.

### Elevated RNase E activity after UV‐C irradiation

**Figure 2:**
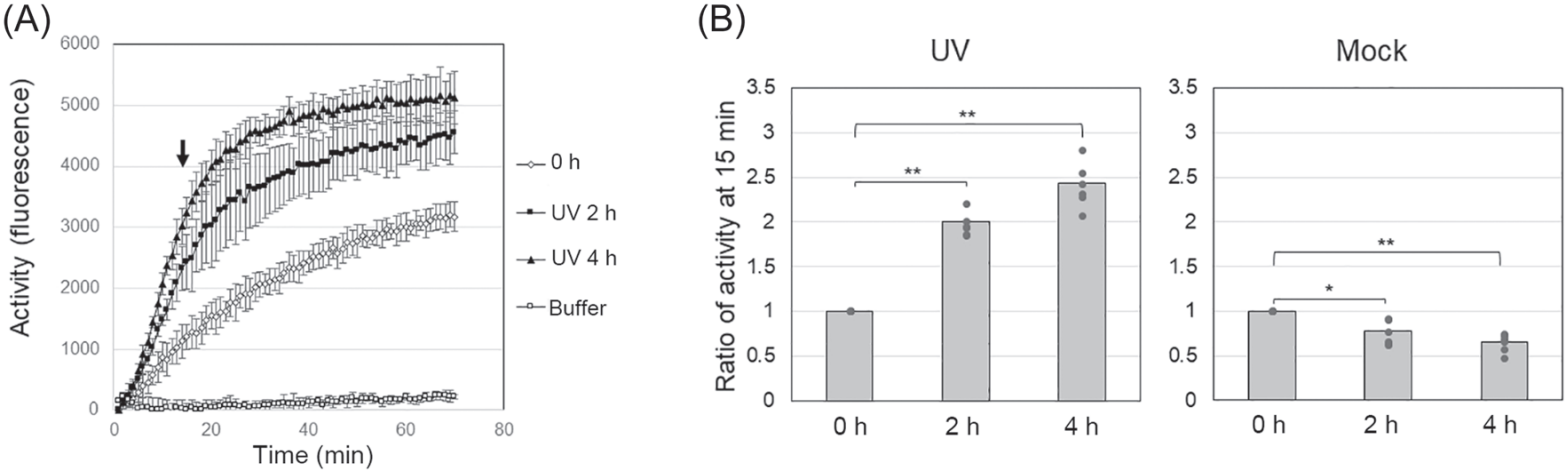
Induction of RNase E activity after UV‐C treatment. (A) RNase E activity in crude cell lysates prepared at the indicated time points after UV‐C treatment (open diamonds: 0 h just after treatment; closed squares: after 2 h; closed triangles: after 4 h; open squares: buffer control) measured in a fluorescence‐based assay with a duration of 70 min. The arrow indicates the high reaction efficiency achieved after 15 min of incubation. (B) Comparison of RNase E activities at 15 min incubation. The values of fluorescence at 15 min incubation were normalized by those before each treatment. The standard deviations of the values obtained for six experiments are shown. Bars represent mean ± SEM (*n* = 6). For statistical evaluation, *p* values were calculated using the paired *t*‐test in Microsoft Excel, *p < 0.05; **p < 0.01.

To determine whether the observed change in *rne* gene expression resulted in higher RNase E enzyme activity, we used a previously established activity assay^50^. This assay is based on a fluorogenic RNA oligonucleotide that consists of a fluorescein amidite (FAM) tag, a Black Hole Quencher 1 (BHQ‐1) quenching tag, and a previously reported *Synechocystis* 6803 RNase E recognition site. In this assay, RNase E activity is monitored via fluorescence from the cleaved FAM oligonucleotide fragment. Using this system, we followed the RNase E activity in *Synechocystis* cells before and after UV‐C treatment. Samples were collected immediately and at 2 and 4 h after irradiation of the cells with UV (400 J/m^2^), and cell extracts were prepared. RNase E cleavage was measured at 1‐min intervals over a period of 70 min using equal amounts of cell lysate protein per sample (Figure 2A). Except in the buffer control, the initial steep increase in fluorescence was followed by a plateau. The reaction efficiency of RNase E obtained by mixing the crude extract with the substrate and incubating for 15 min was compared (Figure 2B). RNase E activity was higher at both measured time points following UV irradiation than in the nonirradiated mock controls (Figure 2B). This result is consistent with the northern blot result, in which substantial accumulation of full‐length *rne* mRNA was observed at 2 h but not earlier (Figure 1C).

### Turnover of RNase E protein after UV irradiation

**Figure 3:**
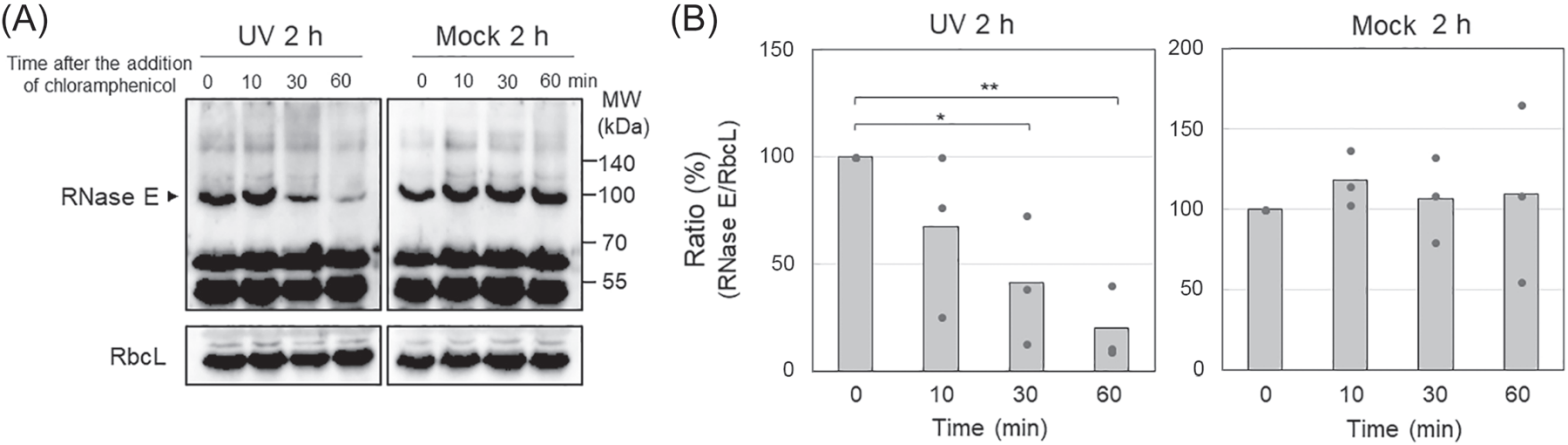
Comparison of the expression level and the stability of RNase E. The translation inhibitor chloramphenicol was added to the cultures 2 h after UV‐C treatment, and cells were harvested at the indicated time points. (A) Western blot analysis. 20 μg of total protein was loaded on an SDS‐PAA gel and subjected to western blot analysis using antibodies against RNase E (arrowhead) or RbcL used as an internal control. (B) Comparison of RNase E protein levels. The signal intensities of RNase E protein (arrowhead) were measured and normalized by those of RbcL protein. Bars represent mean ± SEM (*n* = 3). For statistical evaluation, *p* values were calculated using the paired *t*‐test in Microsoft Excel, *p < 0.05; **p < 0.01.

The observed enhancement of RNase E activity in cell lysates (Figure 2) after UV irradiation could result from the presence of more RNase E protein, higher specific activity of the enzyme, or both. To distinguish these possibilities and to permit the direct detection of RNase E, an antiserum against recombinant *Synechocystis* 6803 RNase E was generated. When used in western blots, this antiserum showed specific signals for RNase E at ~100 kDa, higher than the predicted molecular mass, together with likely nonspecific signals at ~65 and 55 kDa (Figures 3 and S5). The size was further confirmed using an engineered *Synechocystis* 6803 strain (FRE) expressing RNase E with a translationally fused N‐terminal FLAG tag under control of the native promoter (Figure S5A). These observations are consistent with previous results in *E. coli*, in which RNase E of a larger apparent size than predicted was detected in western blot analysis^51^. The fact that RNase E was clearly observed at the protein level (Figures 3 and S5) indicates that the low amounts of its mRNA that are present in the nonstress condition (Figure 1) are sufficient for expression of RNase E protein. For comparison, we used an antiserum against the large subunit of RubisCo and normalized the RNase E signal detected on the immunoblot to the RubisCo signal. This comparison showed that the protein levels of RNase E at 1 and 2 h after UV irradiation were almost the same as those present in the mock condition (Figure S5B), in contrast to the increased accumulation of *rne* full‐length transcripts and upregulated RNase E activity observed 2 h after UV irradiation (Figures 1 and 2). This suggests either that the additional mRNA copies were not efficiently translated or that the stability of the protein was decreased. Thus, we compared the stability of RNase E in cells that had or had not received UV treatment. Chloramphenicol, a translation inhibitor, was added after UV irradiation, and the cells were harvested at the indicated time points. Western blot analysis revealed that the protein levels of RNase E in UV‐treated cells decreased considerably 30 and 60 min after the addition of chloramphenicol, while the RbcL signal and the intensity of the nonspecific bands remained constant (Figure 3). In cells that were not UV‐treated, the intensity of the RNase E signal remained constant despite the addition of chloramphenicol (Figure 3B, mock). We conclude that the observed enhanced RNase E activity was not caused by an increase in the amount of RNase E. We further conclude that active translation of mRNA was required to keep the cellular amount of the enzyme constant following UV treatment and that the decreased stability of RNase E was likely linked to the degradation of UV‐damaged RNase E. These results suggest that after UV irradiation, the turnover of RNase E was accelerated by activation of protein degradation, and resynthesis of the enzyme from the increased amount of *rne* full‐length mRNA.

### Stabilization of *rne* transcripts during the UV stress response

To identify the mechanisms that underlie the increased amount of *rne* full‐length mRNA and hence permit the enhanced turnover of RNase E during the UV stress response, transcript half‐lives were determined. After UV irradiation, rifampicin, which inhibits the initiation of transcription by binding to the β subunit of RNA polymerase^52^, was added to the cultures. For more robust results, the following calculations were based on technical triplicates and biological replicates of the respective two 5′ UTR and ORF segments. The relative amounts of 5′ UTR‐1, 5′ UTR‐2, ORF‐1, and ORF‐2 present at the indicated time points were determined by quantitative real‐time reverse transcription polymerase chain reaction (RT‐qPCR). In parallel, a mock treatment was performed in which UV irradiation was omitted.

**Figure 4:**
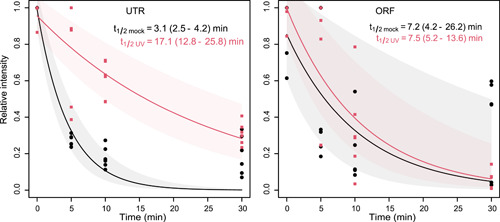
Upregulation of *rne* full‐length transcripts after UV‐C treatment. The relative amounts of *rne* 5′ UTR and ORF transcripts are plotted over time following the addition of the transcription inhibitor rifampicin to cultures after UV‐C treatment for 2 h. The amount of RNA at time point 0 was set to 1.0. Mock‐treated cultures served as controls. The calculated half‐lives of each transcript are indicated. The cells were harvested at the indicated time points for RT‐qPCR analysis using primer sets that anneal to two different regions of the *rne* 5′ UTR (5′ UTR‐1 and 5′ UTR‐2) or the coding region (ORF‐1 and ORF‐2) (Figure S4B,C and Table S2). The data from tests conducted in triplicate were normalized to the amount of 16S rRNA. Half‐life and decay were calculated based on two independent regions in the UTR and the ORF, each with three biological replicates for each region. The fitting curves for the mock treatment and UV stress are given in black and red, respectively. The 95% confidence interval areas are shaded accordingly. RT‐qPCR, quantitative real‐time reverse transcription PCR.

Under the mock conditions, the measured amounts for the probed segments within the 5′ UTR and those within the coding region (ORF)  decreased rapidly (Figure 4). The calculated half‐life of the *rne* 5′ UTR segments was 3.1 min, while that of the ORF was 7.2 min (Table S1), suggesting low stability of the *rne* transcript, similar to *E. coli* ^16^. Compared to the mock condition, substantial stabilization of the 5′ UTR segments but not of the coding region segments was observed after UV stress treatment. The calculated half‐life increased to 17.1 min for the *rne* 5′ UTR, while a half‐life of 7.5 min was determined for the coding sequence (ORF, Figure 4 and Table S1).

Without UV treatment (mock conditions), the stability of the coding region was 2.2 times higher than that of the UTR (Table 1). Therefore, the observed difference in expression levels of the 5′ UTR and the coding region can be explained by premature transcription termination within the 5′ UTR. The calculation of this effect showed that under mock conditions, ~95.9% of the polymerases terminated within the 5′ UTR, while ~65.3% terminated under UV stress (Table 1 and Figure S6). Because the stability (half‐life) of the coding region was roughly unchanged (Table S1), this led to the observed increased intensity levels under UV. Interestingly, the stability of the 5′ UTR under UV stress conditions increased 5.5‐fold (in other words, the decay constant decreased to 0.18) compared to its stability under mock conditions, while the intensity level increased only 2.52 folds, indicating that the rate of transcription of *rne* was reduced by a factor of ~2.2 under UV. Together, the data suggest that the increased *rne* mRNA levels following UV stress were due to reduced premature termination (Figure 7A).

**Table 1:** Change in synthesis rates for the 5′ UTR and ORF regions of the *rne* transcript under mock and UV stress conditions.

	Mock	UV	5**′** UTR
	5′ UTR/ORF	5′ UTR/ORF	UV/mock
*FC* of intensity	11	6.5	2.52
*FC* of decay constant	2.2	0.4	0.18
*FC* of synthesis rate	24.2	2.9	0.46
Termination after UTR	95.9%	65.3%	NA

*FC*, fold change; UTR, untranslated region.

### Detailed analysis of the *rne* 5′ UTR in *Synechocystis* 6803

We observed a particularly large difference in the calculated half‐life of the *rne* 5′ UTR in UV‐treated versus untreated cells (Figure 4) and an abundant accumulation of separate, 5′ UTR‐derived transcripts (Figure 1). Both of these observations suggested that the 5′ UTR plays a pivotal role in the control of transcript stability and the determination of whether full‐length mRNA will accumulate. These findings led us to hypothesize that RNase E expression may be regulated via its extremely long 5′ UTR. Recent mapping of RNase E‐dependent cleavage sites in *Synechocystis* 6803 by TIER‐seq, which also points at sites within the *rne* 5′ UTR, supports this idea^36^. However, in the TIER‐seq analysis, mainly 5′ ends were mapped, whereas the 3′ ends remained largely unexplored.

**Figure 5:**
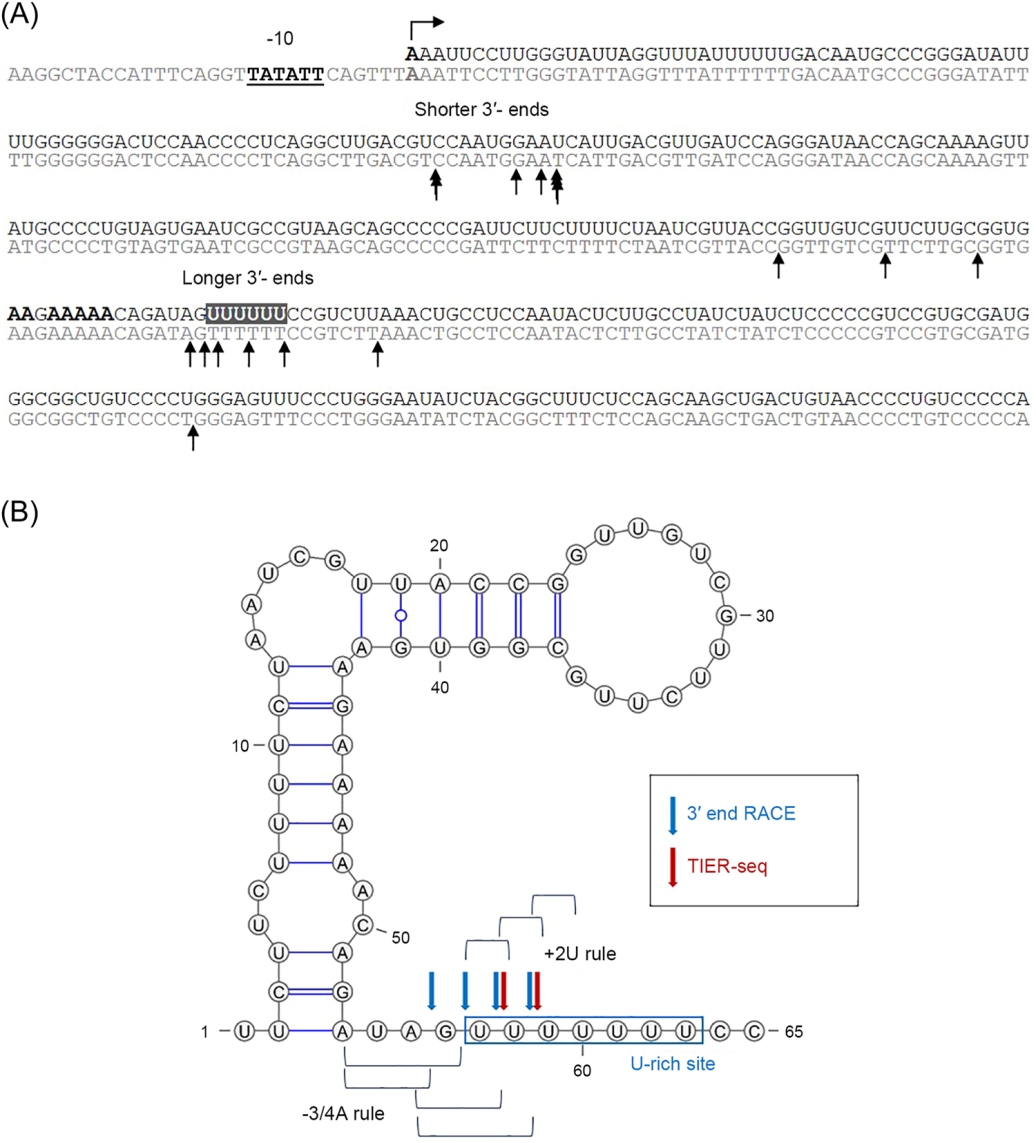
Analysis of 3′ ends within the *rne* 5′ UTR. (A) The 3′ ends mapped by the 3′ RACE assay within the first 355 nt of the *rne* 5′ UTR are indicated by arrows (overlapping arrows if the same end was found several times). The promoter (−10), TSS, and A‐rich sites are represented by boldface letters. The U‐rich site is indicated by the gray box and white characters. The 3′ ends mapped primarily to two regions that were located 78–90 nt (shorter 3′ ends) and 214–229 nt (longer 3′ ends) downstream from the TSS of *rne*. (B) Secondary structure of the U‐rich site that forms part of the *rne* 5′ UTR predicted by RNAfold on the ViennaRNA website^53^ with default settings and visualized using VARNA version 3.93^54^. RNA 3′ ends mapped by 3′ RACE and 5′ ends mapped by TIER‐seq^36^ are indicated by blue and red arrows, respectively. The RNase E consensus sequence suggested by TIER‐seq (+2U rule: uridine at 2 nt downstream of the cleavage site; −3/4 A rule: adenine at 3–4 nt upstream) is also shown.

Therefore, we conducted 3′ RACE analysis to map possible 3′ ends within the *rne* 5′ UTR and sequenced the complementary DNA (cDNA) inserts of 17 clones. These 3′ ends primarily mapped to two regions that are located 78–90 nt (shorter 3′ ends) and 214–229 nt (longer 3′ ends) downstream of the TSS (Figure 5A). The most prominent RNase E cleavage sites within the *rne* 5′ UTR mapped by TIER‐seq^36^ also lie within the U‐rich region (228 and 229 nt downstream the TSS; Figure 5B), as well as further in the 3′ direction (237 and 320 nt from the TSS). Moreover, compared to the shorter 3′ ends, the sequence surrounding the longer 3′ ends of the *rne* 5′ UTR is well conserved in *Synechocystis* 6803 and *Synechocystis* 6714 (Figures S8 and 5A). These observations further support the hypothesis that the expression of RNase E is self‐regulated by cleavage within the 5′ UTR, with a focus on the AU‐rich region.

**Figure 6:**
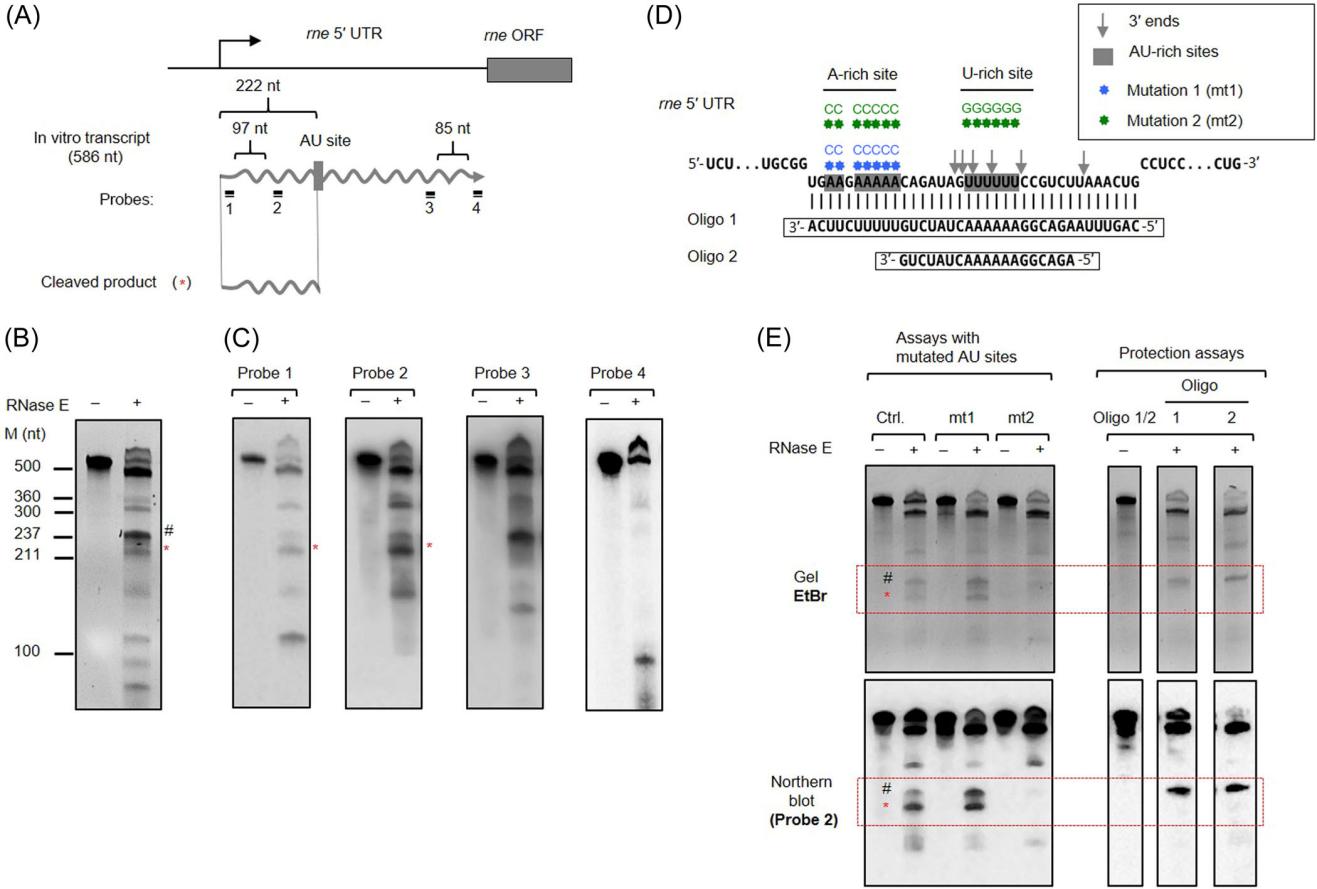
In vitro RNase E cleavage assay. (A) Summary of the cleavage of the *rne* 5′ UTR transcript by RNase E recombinant protein. The *rne* 5′ UTR transcript and the major products detected in the assay are shown, together with the locations at which probes that recognize different regions within the *rne* 5′ UTR transcript hybridize (Figure S4D and Table S2). (B) In vitro transcripts of the *rne* 5′ UTR were incubated with (+) and without (−) recombinant *Synechocystis* RNase E, and the resulting RNA cleavage patterns were visualized by ethidium bromide staining. Fragment sizes were estimated using NEB ssRNA markers. The major bands generated by RNase E digestion are marked by red asterisk and hash symbol. The red asterisk marks a longer fragment that is similar in length to a fragment that could extend from the TSS to the two major 3′ ends as determined by 3′ RACE (Figure 5). (C) Northern blot analysis of RNase E‐digested *rne* 5′ UTR transcripts. In vitro transcripts of the *rne* 5′ UTR were incubated with (+) and without (−) recombinant *Synechocystis* RNase E. After separation of the digestion products on PAA gels and blotting, the membranes were hybridized with specific probes (Figure S4D and Table S2). (D, E) In vitro RNase E cleavage assays using mutant *rne* 5′ UTR transcripts and protection of the transcripts from RNase E attack. (D) Scheme of point mutations within the *rne* 5′ UTR transcript and the sequences of oligo‐RNAs used in the protection assay. The 3′ ends of the *rne* 5′ UTR, mapped by 3′ RACE, are indicated by gray arrows. (E) RNase E cleavage assay (left) and protection assay (right). The RNA cleavage patterns were visualized on ethidium bromide (EtBr)‐stained 7 M urea–6% PAA gels (upper image) and analyzed by northern blot hybridization using probe 2 (lower image). The specific bands generated by RNase E digestion are marked by the same asterisk and hash symbols.

In *Synechocystis*, not only RNase E but also RNase J, which can function as an endoribonuclease, are active^23^. To discriminate between these two enzymes and to gain further insight into the location of cleavage sites within the 5′ UTR, RNase E in vitro cleavage assays were performed. The complete *rne* 5′ UTR of 583 nt (plus 3 nt resulting from the initiation of transcription by the T7 RNA polymerase generating the in vitro transcript; Figure 6A) was transcribed in vitro and incubated with purified recombinant RNase E, and the cleavage products were separated by polyacrylamide (PAA) gel electrophoresis. After treatment with RNase E, several distinct RNA fragments were obtained, confirming the presence of multiple RNase E sites within the *rne* 5′ UTR (Figure 6B). Next, we performed northern blot analysis to identify the major fragments generated by cleavage within the *rne* 5′ UTR (Figure 6C). We used probes that recognize either the 5′ or the 3′ parts of the *rne* 5′ UTR with respect to the cleavage site (Figure S4D). A particular fragment (labeled in Figure 6C by asterisks) resulting from the RNase E cleavage was noted; as this fragment was observed with probes 1 and 2, but not with probes 3 or 4, it must have derived from the first part of the 5′ UTR (Figure 6C). The length of this fragment corresponds well to that of an RNA that could extend from the TSS to the AU‐rich site.

To unambiguously demonstrate that the observed cleavage was performed by RNase E, we focused on the AU‐rich region, to which the mapped 3′ ends of *rne* 5′ UTR subfragments had pointed and that is conserved in *Synechocystis* 6803 and *Synechocystis* 6714 (Figure S8). We tested the effects of point mutations in the AU‐rich region (Figure 6D). A‐to‐C substitutions at the A‐rich site had no effect, while the cleavage product was no longer observed when nucleotides were substituted at both the A‐rich and the U‐rich sites (Figure 6E). However, these substitutions together should also have led to a different secondary structure. Therefore, we performed protection assays using oligo RNAs that cover either only the U‐rich site or both the A‐rich and the U‐rich sites. The cleavage product in question disappeared after the addition of either of the two tested oligo RNAs, both of which cover the U‐rich site (Figure 6E). Oligo 2 was shorter than oligo 1 and did not cover the A‐rich site, which hence had made no difference. These results indicated that the U‐rich site is a preferred site for RNase E cleavage in the *rne* 5′ UTR in vitro and are consistent with the results of our analysis of RNA ends in vivo by 3′ RACE mapping (Figure 5B) and with the results of 5′ end mapping according to the TIER‐seq data set^36^.

## DISCUSSION

**Figure 7:**
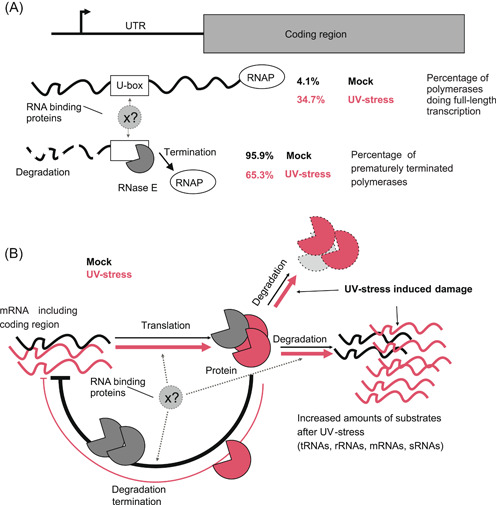
Hypothetical model of the regulation of RNase E expression in *Synechocystis* 6803. (A) The 5′ UTR of the *rne* message contains an RNase E‐sensitive U‐box. The (initial) endonucleolytic cleavage by RNase E destabilizes the UTR and might also lead to termination of transcription. Regardless of the exact molecular mechanism, termination is the main factor that accounts for differences in expression of the 5′ UTR and the coding region. Additional factors might be involved in these processes. In the absence of UV stress, 95.9% of all RNA polymerase molecules terminate at a point before the coding region, and only 4.1% transcribe the full‐length message. Under UV stress conditions, the percentage of prematurely terminating polymerases decreases to 65.3%. (B) Hypothetical model of the autoregulatory negative feedback loop. Under normal growth conditions, the number of *rne* transcripts is maintained at a low level by a combination of RNA cleavage and transcription termination. When UV stress occurs, the number of alternative RNase E targets increases, and the stability of the RNase E protein is reduced. As a result, less RNase E activity is allocated to its own UTR, the autoregulation is relieved, and the system reaches a new equilibrium in which the concentrations of RNase E mRNA and protein are higher.

Here, we demonstrate that the UV stress response in *Synechocystis* 6803 involves comprehensive transcriptome remodeling and accelerated RNase E turnover and provide evidence for a regulatory mechanism underpinning this process. The results of in vitro RNase E assays in the presence and absence of protecting oligonucleotides and with RNAs containing specific point mutations (Figure 6) are consistent with the results of 3′ end RACE mapping and 5′ end mapping by TIER‐seq, confirming that RNase E cleaves within the 5′ UTR of its own mRNA in the U‐rich site (Figure 5). Moreover, the TIER‐seq data indicate the possible presence of additional sites in the region approximately 10–100 nt downstream of the U‐rich site^36^. These facts suggest that RNase E regulates its level of expression by targeting the 5′ UTR of its own transcript. Superficially, these results resemble the autoregulation of RNase E levels in *E. coli* ^16^, ^19^. However, the observed increase in the full‐length *rne* transcript level after UV stress cannot be fully explained by stability control of the *rne* 5′ UTR. Our data suggest that autoregulation depends on the premature termination of *rne* transcripts and does not involve an increased transcription rate (Figures 7A and S6). Under standard growth conditions, it seems to be uneconomical for the cell to stop most transcription at the *rne* 5′ UTR. However, such a mechanism could be advantageous as it permits the rapid upregulation of RNase E production if needed.

The RNase E‐mediated cleavage rate at the 5′ UTR site might be influenced by an abundance of alternative substrates in the cell or due to protection by an RNA‐binding protein (Rbp). The identification of such hypothetically involved Rbp is an interesting topic for future research.

Our proposed model for the feedback regulation of *Synechocystis* RNase E is shown in Figure 7B. Under nonstress conditions, the number of *rne* transcripts remains at a low level. This appears to be regulated by cleavage within the *rne* 5′ UTR U‐rich region, as well as by termination of transcript elongation. UV irradiation likely damages nucleic acids as well as proteins, including RNase E. UV‐induced RNA‐protein crosslinks can lead to stronger damage of Rbps, including the endoribonuclease RNase E. As a consequence, the degradation of UV‐damaged RNase E protein should become selectively accelerated. In addition, we assume that the presence of UV‐damaged RNAs drastically increases the substrate pool for RNase E. Both processes lead to a scenario in which less RNase E activity can be allocated to its own mRNA, leading to a more stable 5′ UTR and reduced transcription termination before the ORF. As a result, more active RNase E is synthesized.

A similar control mechanism might also exist in other cyanobacteria because extremely long *rne* 5′ UTRs were mapped in several different species (Figure S1A). An additional regulatory mechanism was reported in the marine cyanobacterium *Prochlorococcus* sp. MED4, in which RNase E levels were found to increase during lytic infection by the cyanophage P‐SSP7; the increase in RNase E levels may support phage replication by generating a source of nucleotides from stimulated RNA degradation^55^, ^56^. During phage infection, *rne* transcription proceeds from an alternative TSS, resulting in a shorter mRNA variant that lacks the regulatory 5′ UTR^56^. These observations suggest that there is additional diversity in the control mechanisms of RNase E in cyanobacteria. While the mechanism described here ensures the presence of a certain level of RNase E, it does not explain the increased enzymatic activity that we observed 2 and 4 h after UV irradiation. Because there was no concomitant increase in the amount of RNase E protein, either the specific activity of the enzyme is enhanced by a regulatory factor or by protein modification or the fraction of active enzyme is higher among freshly synthesized enzymes than among the existing pool.

Our microarray analysis revealed changes in transcriptome composition after UV treatment over time, and these changes showed similarities to and differences from those observed in other bacteria. When DNA is damaged by UV light, an SOS response is triggered, leading to DNA repair and the rebuilding of cellular components; this is accomplished by pausing cell division and energy production. Consistent with this widely conserved mechanism, we observed transient repression of genes involved in translation and energy metabolism one hour after UV irradiation of *Synechocystis* 6803. On the other hand, no significant induction of SOS gene homologs, such as *lexA*, *recA*, *uvrA*, *uvrB*, and *uvrC*, occurred; instead, a marked induction of *rne* and *rnj* was observed (Figures 1 and S3I). In *E. coli*, SOS‐responsive genes were not induced in mutants of *rne* and *rng* (the latter encodes the RNase G, a homolog of RNase E), suggesting that RNase E is involved in the control of the SOS response^57^. While the cellular response to UV has been studied in *Synechocystis* 6803 at the protein level^58^, the regulatory mechanisms involved, including those that control the SOS response, are not clear in cyanobacteria. The functions of homologs of the *E. coli*‐type SOS response regulator LexA are not conserved in several species of cyanobacteria^59^. LexA has been reported to be unrelated to the regulation of the SOS response in *Synechocystis* 6803^39^, ^60^, in which it instead functions as a transcriptional regulator of fatty acid metabolism^61^ and salt stress response^62^. In addition, transcript levels of the *recA* gene, which plays a major role in DNA repair and recombination, are negatively regulated by UV light and oxidative stress at the posttranscriptional level^39^ through a mechanism in which both RNase E and RNase J are thought to be involved. Further study of the posttranscriptional regulatory mechanisms involved in the cyanobacterial SOS response, including those related to RNase E, is needed.

In *Synechocystis* 6803, it has been reported that RNase E binds to a short hairpin RNA structure and then cleaves the RNA in an AU‐rich region immediately downstream of the hairpin. Such sites have been described within the 5′ UTR of *psbA2*, upstream of *crhR* ^29^, ^34^, and in maturation of crRNAs from long precursor RNA in a CRISPR–Cas subtype III‐Bv system^35^. Systematic mapping of RNase E sites in *Synechocystis* 6803, moreover, points to the frequent presence of a uridine 2 nt downstream of the cleavage site (+2U rule) and an adenine 3 or 4 nt upstream (−3/4A rule); together, these residues are capable of forming an “AU clamp”^36^. We observed a very similar architecture of the U‐rich site determined here (Figure 5B).

Further studies targeting proteins that may bind to the long *rne* 5′ UTR or modulate RNase E activity will be necessary to elucidate the detailed mechanism of the selective posttranscriptional upregulation of *rne* transcripts during the UV stress response. The RNA chaperone Hfq modulates regulatory sRNA and target RNA structures and their interactions with each other^63^; however, there is no evidence that Hfq binds RNA in *Synechocystis* 6803^64^, ^65^. Another type of Rbp containing a single RNA recognition motif is widely conserved in cyanobacteria^66^, ^67^, ^68^, ^69^ and even in plant chloroplasts^70^. In *Synechocystis* 6803, Rbp2 and Rbp3 are involved in the regulation of photosynthetic gene expression and thylakoid membrane targeting through transcript binding^71^. In our study, the expression of these *rbp* genes was found to be induced at the same time as the expression of *rne* in response to UV stress (Figure S3K–M), suggesting the involvement of Rbps in the UV stress response.

In *E. coli*, RNA degradation and processing by RNase E are known to be modulated by RNase E‐binding proteins such as RraA, RraB, and RapZ^20^, ^72^, ^73^, ^74^; however, no homologs of RraA or RraB have been characterized in cyanobacteria. Homologs of RapZ exist in certain cyanobacteria (*Synechococcales*, *Leptolyngbya*, *Gloeobacter*, and others) but are not conserved in *Synechocystis*, and their function is still unknown. In the cyanobacterium *Synechococcus elongatus* PCC 7942, RNase E activity was inhibited when the DnaK2 and DnaJ2 chaperones were added to in vitro assays^75^. Thus, these chaperones clearly have the potential to impact RNase E activity.

RNase E is selectively degraded after UV irradiation; however, the involved mechanism is unknown. The identification and functional characterization of proteins that interact with RNase E or its 5′ UTR is a promising topic for future research and should lead to a better understanding of the feedback regulation of RNase E and regulatory circuits within the RNA metabolism of cyanobacteria.

## MATERIALS AND METHODS

### Bacterial strains and growth conditions

*Synechocystis* sp. PCC 6803 PCC‐M strain^76^ was grown photoautotrophically (40 µmol photons/m^2^/s) at 30°C in BG‐11 medium^77^ supplemented with 20 mM HEPES‐NaOH (pH 7.5).

### UV irradiation and viability assay

For viability testing, exponentially growing cells at a density of 2 × 10^7^ cells/ml were transferred to plastic dishes without lids and irradiated with UV‐C (254 nm) using UV lamps (UVP Inc.) at a dose of 400–16,000 J/m^2^. The cells were collected and then spread on solid medium before or after UV irradiation. Surviving colonies were counted after 7 days of growth. Consistent with previous reports^39^, this analysis revealed a dose of 400 J/m^2^ as optimal, which therefore was used in all subsequent assays.

### RNA extraction and northern blot analysis

At the indicated times after UV‐C irradiation (400 J/m^2^ or mock treatment for 200 s), *Synechocystis* 6803 cells were harvested and total RNA was extracted as described previously^78^, ^79^. The extracted RNA samples were analyzed by electrophoretic separation of 3 μg of RNA on 1% agarose‐urea gels and electroblotted onto Hybond‐N + membranes (Amersham). For northern blot hybridizations shown in Figure 1C, the PCR‐generated probe templates were obtained using the primers slr1129‐5UTR‐f and slr1129‐5UTR‐rT7 (*rne* 5′ UTR probe) or slr1129orf‐f and slr1129orf‐rT7 (*rne* ORF probe); for the primer sequences, see Table S2 and Figure S4A. The single‐strand transcript probes were generated by in vitro transcription from these templates using the MAXIscript kit (Ambion) as described^35^. The membranes were prehybridized for 60 min at 62°C with hybridization buffer (Roche) in glass tubes under continuous rotation. The single‐stranded labeled RNA probes were added to the prehybridized membranes for hybridization at 62°C overnight. The membranes were washed at 57°C with wash buffer I (2× SSC, 1% SDS), II (1× SSC, 0.5% SDS), and III (0.1× SSC, 0.1% SDS) for 10 min each.

The oligonucleotide probes used in Figure 6C were 5**′** labeled using 30 μCi [γ‐^32^P]ATP, 5 µl oligonucleotide (10 pmol/µl), and T4 polynucleotide kinase (Thermo Fisher Scientific) as previously described^79^. Membranes were prehybridized for at least 30 min at 45°C with hybridization buffer (Roche) in glass tubes under continuous rotation. The labeled oligonucleotide probes were placed on ice and then added to the prehybridized membrane for hybridization at 45°C overnight. Membranes were washed at 40°C with wash buffers I, II, and III as above, for 10 min each. A BIO‐RAD Molecular Imager FW system was used for detecting the signals on a Kodak phosphorimaging screen. Quantity One software (BIO‐RAD) for signal processing.

### Microarray analysis

The here used Agilent microarrays contain oligonucleotide probes representing all annotated mRNAs as well as most other expressed transcripts, allowing precise determination of individual transcripts with respect to both DNA strand and genomic location. We used previously described conditions for hybridization and analysis^80^. Total RNA (5 µg) was extracted from *Synechocystis* 6803 cells collected 1 or 2 h after UV‐C irradiation at 400 J/m^2^ or following mock treatment and directly labeled with Cy5 (without cDNA synthesis) using the ULS labeling kit (Kreatech Diagnostics) according to the manufacturer's protocol. RNA fragmentation and hybridization for Agilent one‐color microarrays were performed according to the manufacturer's instructions using 1.65 µg of labeled RNA in biological duplicates. The microarrays were scanned with an Agilent microarray scanner C, model G2505C, using Agilent Scan Control and Feature Extraction 10.7.3.1 software. The raw data were quantile normalized using limma R software as part of Bioconductor version 3.16. The differences in the transcriptomes of cells subjected to UV‐C and mock conditions were determined for each time point. A transcript was considered differentially expressed when it met the significance criteria (log_2_ *FC* ≥ │1│, adj. *p* ≤ 0.05). *p* values were adjusted for multiple testing using the Benjamini–Hochberg method. The comparative microarray data are shown in Data S1, and the raw data have been deposited in the GEO database under the accession number GSE186330.

### Estimation of transcript half‐lives by RT‐qPCR

We designed specific primer sets for use in analyzing two segments within the *rne* 5′ UTR (5′ UTR‐1 and 5′ UTR‐2) and two segments within the coding region of the RNA (ORF‐1 and ORF‐2) by RT‐qPCR (Figures S4B and S4C). To estimate the half‐lives of the *rne* 5′ UTR and coding region segments, 200 µg/ml rifampicin, a transcription inhibitor^52^, was added 2 h after UV‐C treatment of the cells at 400 J/m^2^. RNA samples were prepared from cells collected 0, 5, 10, and 30 min after rifampicin addition by rapid filtration of the cells onto Supor 800 membranes as described above, and relative amounts of the RNA were quantified by RT‐qPCR. For this, cDNA was prepared from 2 µg of each RNA sample using the PrimeScript II 1st strand cDNA synthesis kit (TaKaRa) with 40 units of RNasin Plus RNase inhibitor (Promega) according to the manufacturer's instructions. RT‐qPCR was performed using a StepOnePlus real‐time PCR System (Applied Biosystems) in standard mode (10 min at 95°C, followed by 40 cycles of 15 s at 95°C, 15 s at 55°C, and 60 s at 72°C). Each 20 μl reaction contained 10 μl of Power SYBR Green Mix (Applied Biosystems), 2 μl of cDNA, and 0.4 μl each of the forward and reverse primers (final concentration 200 nM). The primers (Table S2) were synthesized by Eurofins MWG Operon. All reactions were conducted in triplicate, and 16S rRNA was amplified as a reference. Melting curves for the amplifications showed only single products. The data were analyzed using the StepOnePlus system SDS software (Applied Biosystems) with manual *C*_t_ and automatic baseline settings. Relative transcript quantities were calculated using the ΔΔ*C*_t_ method. The RT‐qPCR data were used to calculate transcript half‐lives by fitting the decay time‐course abundance curves to an exponential decay function. The degradation constants and half‐lives were calculated by fitting the data to an exponential decay curve with the R nls function.

### Calculation of synthesis rates

Assuming steady‐state expression of a given transcript, the transcript levels (*Int*) are defined by the ratio of its synthesis rate to its transcription rate (*α*) and the degradation constant (*λ*), as follows:

$$\mathrm{Int}=\frac{\alpha }{\lambda }$$

The fold change (*FC*) in the expression of two transcripts (*i,j*) is

$${\mathrm{FC}}_{i,j}=\frac{{\mathrm{Int}}_{i}}{{\mathrm{Int}}_{j}}=\frac{\alpha_{i}}{\lambda_{i}}\times \frac{\lambda_{j}}{\alpha_{j}}$$

With the use of the microarray intensity data and the fits for the degradation constants, we can calculate the ratio of synthesis rates (*FCsynt*).

$${\mathrm{FCsynt}}_{i,j}=\frac{\alpha_{i}}{\alpha_{j}}=\frac{{\text{Int}}_{i}}{{\text{Int}}_{j}}\times \frac{\lambda_{i}}{\lambda_{j}}$$

Assuming a single promoter for *rne* transcription, a synthesis ratio $FCsynt_{\mathrm{UTR},ORF}>1\;$indicates a termination after the 5′ UTR. Comparing the synthesis ratios of the UTR under mock and UV stress conditions, ${\mathrm{FCsynt}}_{\mathrm{UV},\mathrm{mock}}<1\;$indicates a reduced transcription rate under UV stress conditions.

### Fluorogenic cleavage assay

A fluorogenic cleavage assay was performed as described previously^50^, with modifications in preparing the cell extract. An RNA oligonucleotide with a FAM tag and a BHQ‐1 quenching tag containing the RNase E recognition site within  the *psbA2* 5′ UTR was used. Cleavage within the fluorogenic oligonucleotide separates the two tags from each other, which can be measured by the increase in fluorescence. After UV irradiation at a dose of 400 J/m^2^, the cells were collected immediately or after cultivation at room temperature for 2 and 4 h and were then stored at −20°C. The cell pellets were resuspended in 300 µl of reaction buffer (25 mM Tris‐HCl [pH 8.0], 60 mM KCl) in a vial containing 300 mg of glass beads and broken in a Mini‐BeadBeater‐16 (BioSpec). The resulting cell extract was centrifuged at 400*g* for 5 min at 4°C. The supernatant containing the soluble and membrane proteins was transferred to a new tube and used in the assay. The cleavage assays were carried out at 30°C in a 50‐µl reaction mixture containing reaction buffer (25 mM Tris‐HCl (pH 8.0), 60 mM KCl) with 5 mM MgCl_2_, 100 mM NH_4_Cl, 0.1 mM DTT, 5% (w/v) glycerol, 0.06 µM fluorogenic RNA, and 50 µg of crude extracts prepared in the above conditions. Finally, 20 µl of RNase‐free immersion oil was added to each sample to avoid evaporation. The cleavage reaction was monitored in technical triplicates for 70 min at 1‐min intervals by fluorometry using a Victor^TM^ X^3^ multilabel plate reader (PerkinElmer; excitation at 480 nm, emission at 520 nm).

### Overexpression and purification of *Synechocystis* 6803 RNase E and preparation of polyclonal antisera

Recombinant RNase E was expressed and purified as described^29^. The preparation of rabbit antiserum against the purified RNase E protein was based on previous methods^81^ (Protein Purify Co. Ltd.). The recombinant RNase E was subjected to SDS–PAA gel electrophoresis and recovered from the gel. After crushing and mixing with adjuvant, the gel slices were injected into rabbits. Each antigen was injected five times over a period of 3 months, and the resulting antibody titers were measured by ELISA. Centrifugation was used for the preparation of whole antiserum from the collected blood samples. The antiserum was stabilized by the addition of NaN_3_ at a final concentration of 0.1% (w/v) and stored at −80°C until use.

### Western blot analysis and determination of RNA half‐life

Crude extracts of the cells were carried out as previously described, with minor modifications^82^. The *Synechocystis* 6803 FRE strain, which expresses an N‐terminally FLAG‐tagged version of RNase E from its native chromosomal site, was used for size estimation of RNase E. To avoid degradation of RNase E protein, the crude extracts were prepared immediately after cell harvesting. After UV‐C irradiation at 400 J/m^2^ or mock treatment, *Synechocystis* 6803 cells were harvested by centrifugation and used for the preparation of crude protein samples as previously described^82^. Twenty micrograms of each sample was analyzed by western blot analysis using primary antisera against RNase E, FLAG‐tag (No. F3165; Sigma‐Aldrich), and RbcL (No. AS03 037; Agrisera) at dilutions of 1:3000, 1:10,000, and 1:8000, respectively; HRP‐conjugated anti‐mouse (for FLAG‐tag, No. NA931V) and rabbit IgG (for RNase E and RbcL, No. NA934V) (GE Healthcare) were used as the secondary antibody. The images and quantities of each protein signal were obtained using a ChemiDoc XRS + system with Image Lab software (Bio‐Rad laboratories).

To estimate the half‐life of RNase E protein, 250 µg/ml of chloramphenicol was added 2 h after UV‐C treatment of the cells at 400 J/m^2^. Crude extracts were prepared from the cells as described above and separated by SDS–PAA gel electrophoresis, followed by western blot analysis.

### Rapid amplification of 3′ ends

Rapid amplification of 3′ ends (3′ RACE) was performed according to Argaman et al^83^. Total RNA was prepared from a *Synechocystis* 6803 culture grown under standard conditions. After ligation of an RNA adapter, the RNA was reverse‐transcribed and PCR‐amplified using primers that anneal to the 5′ UTR of *rne* or to the adapter sequence. An electrophoresis gel image of PCR products is shown in Figure S7. DNA fragments were ligated into the pGEM‐T vector and transferred into *E. coli*. Single colonies were picked, and the inserts were sequenced by Sanger sequencing. The sequences of the RNA adapters and DNA primers used in this study are listed in Table S2.

### In vitro cleavage assay

The *rne* 5′ UTR was transcribed in vitro from a PCR‐generated template using the oligonucleotides rne5UTR‐T7‐fw and rneATG‐rev. For in vitro transcription of the variants of the *rne* 5′ UTR (mutation 1 and mutation 2, Figure 6E), template DNA was synthesized in a fusion‐PCR approach. Briefly, *rne* 5′ UTR fragments 1, 2, and 3 were amplified using the oligonucleotide pairs rne5UTR‐T7‐fw/frag1‐rev, frag2‐fw/rneATG‐rev, and frag3‐fw/rneATG‐rev, respectively. Next, fragments 1 and 2 (fusion product 1) and fragments 1 and 3 (fusion product 2) were combined and used as template DNA for fusion PCR, each in combination with oligonucleotides rne5UTR‐T7‐fw and rneATG‐rev. Fusion products 1 and 2 were used as template DNA for in vitro transcription of *rne* 5′ UTR mutation 1 and mutation 2 variants, respectively. Residual template DNA was depleted as described^50^, and the full‐length RNAs generated in vitro were purified from PAA gels as described^31^. In vitro RNase E cleavage assays were performed as described previously^50^ with the following modifications: 0.8 pmol of RNA was incubated with 7 pmol of *Synechocystis* 6803 recombinant RNase E for 30 min at 30°C; after separation of the mixture on 7 M urea‐6% PAA gels, RNA was transferred to a Hybond‐N nylon membrane (Amersham) and subjected to northern blot hybridization. RNA gel blot hybridizations, 5′‐radiolabeling, and purification of oligonucleotide probes were performed as described previously^50^. The oligonucleotides used as probes are listed in Table S2.

### RNase E protection assay

Reaction mixtures (3 µl each) containing 0.5 pmol of in vitro transcribed RNA (*rne* 5′ UTR, *rne* 5′ UTR mutation 1, and *rne* 5′ UTR mutation 2) and 2 pmol of the oligonucleotides as‐rne 210/228 and as‐rne 200/234 were incubated for 5 min at 85°C and then briefly chilled on ice. The reaction mixture was then supplemented with 1 µl 5× RNase reaction buffer (125 mM Tris‐HCl (pH 8.0), 300 mM KCl, 25 mM MgCl_2_, 500 mM NH_4_Cl, 0.5 mM DTT) and incubated at room temperature for 15 min. Recombinant RNase E (7 pmol) was added to increase the total reaction volume to 5 µl, and incubation was continued at 30°C for 15 min. RNase E activity was quenched by the addition of 1 µl of 0.5 M EDTA and 1× volume loading buffer. Following heating at 95°C for 3–5 min, cleavage products were separated on 7 M urea–6% PAA gels and subjected to northern blot hybridization as described above.

## AUTHOR CONTRIBUTIONS

Satoru Watanabe performed the majority of biochemical and molecular analyses in *Synechocystis* 6803. Damir Stazic and Claudia Steglich performed in vitro assays with RNase E. Jens Georg performed statistical analyses of RNA stability. Shota Ohtake, Yutaka Sakamaki, and Megumi Numakura performed RT‐qPCR and western blot analysis. Munehiko Asayama generated the antiserum against RNase E. Taku Chibazakura and Annegret Wilde analyzed the data with Satoru Watanabe and Wolfgang R. Hess. Satoru Watanabe and Wolfgang R. Hess drafted the manuscript with input from all authors. All authors read and approved the final manuscript.

## ETHICS STATEMENT

There is no work on animals or human subjects in this study.

## CONFLICT OF INTERESTS

The authors declare no conflict of interests.

## Supporting information

Supporting information.

Supporting information.

Supporting information.

## Data Availability

Microarray data have been deposited with the Gene Expression Omnibus (GEO) data base with the accession number GSE186330.
