# Supplementary material for: Regulation of RNase E during the UV stress response in the cyanobacterium Synechocystis sp. PCC 6803
Source: mLife. 2023 Feb 15;2(1):43–57. doi: 10.1002/mlf2.12056 (PMC10989929; doi:10.1002/mlf2.12056)
Supplement: Supplementary file 1 — Supporting information. [file MLF2-2-43-s003.pdf]

Supporting Information

Regulation of RNase E during the UV-stress response in the cyanobacterium *Synechocystis* sp. PCC 6803

Satoru Watanabe[1], Damir Stazic[2,3], Jens Georg[2], Shota Ohtake[1], Yutaka Sakamaki[1], Megumi Numakura[1], Munehiko Asayama[4], Taku Chibazakura[1], Annegret Wilde[5], Claudia Steglich[2], and Wolfgang R. Hess[2†]

Authors' affiliations:
[1]Department of Bioscience, Tokyo University of Agriculture, 1-1-1 Sakuragaoka, Setagaya-ku, Tokyo 156-8502, Japan
[2]Faculty of Biology, Genetics and Experimental Bioinformatics, University of Freiburg, Freiburg, D-79104 Freiburg, Germany
[3]Present address: Nexxiot, Prime Tower (Hardstrasse 201), 8005 Zürich, Switzerland
[4]School of Agriculture, Molecular Genetics, Ibaraki University, 3-21-1 Ami, Inashiki, Ibaraki 300-0393, Japan
[5]Faculty of Biology, Molecular Genetics, University of Freiburg, D-79104 Freiburg, Germany

Contents:

- Figure S1-S8
- Table S1-S2
- The legends of Supplementary Data 1 and 2

Supplementary Figure S1

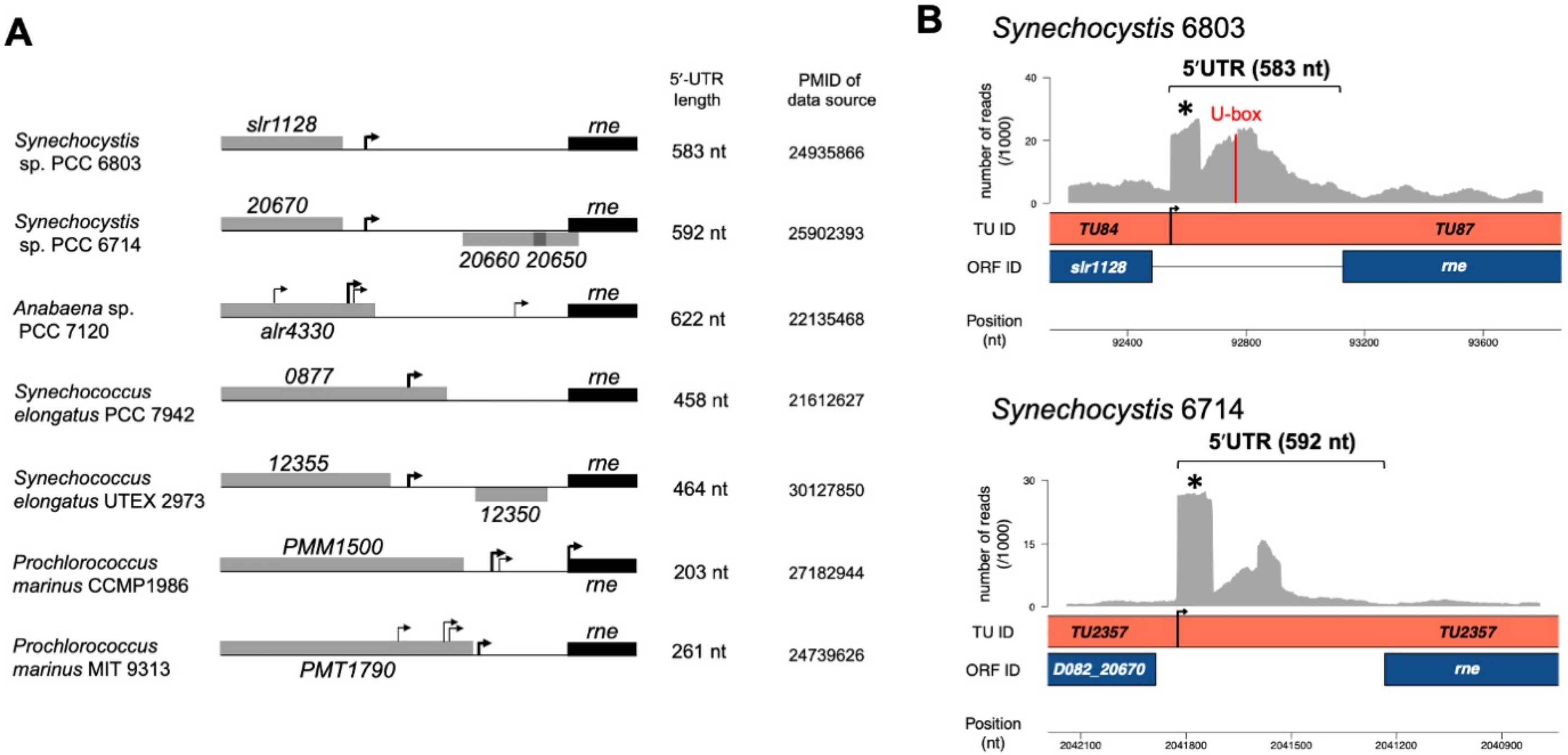

Figure S1. Long 5′ UTRs of the *rne* gene in cyanobacteria. (A) Comparison of *rne* 5′ UTRs in different cyanobacteria. Transcription start sites (TSSs, arrows) and upstream regions of the *rne* in several species are illustrated, as is the length of the *rne* 5′ UTR. Information on the TSS in each organism was obtained from comprehensive experimental data, including TSS-seq analysis. The bold arrows indicate major TSSs. (B) The *rne* genomic locus in *Synechocystis* 6803 and *Synechocystis* 6714 with coverage by RNA-seq data extracted from previous analyses (49). Protein-encoding genes are shown in blue, and transcription units (TUs) are shown in red. The graphs shown in gray represent the coverage by RNA-seq reads. The y-axes of the graphs indicate square-root-scaled coverage values, and the x-axes show the chromosomal position in bp. A red bar indicates the location of the U-box in the *rne* 5′ UTR. Asterisks indicate the highly expressed *rne* 5′ UTRs in *Synechocystis* 6803, annotated as sRNA *ncr0020* (Figure 1B).

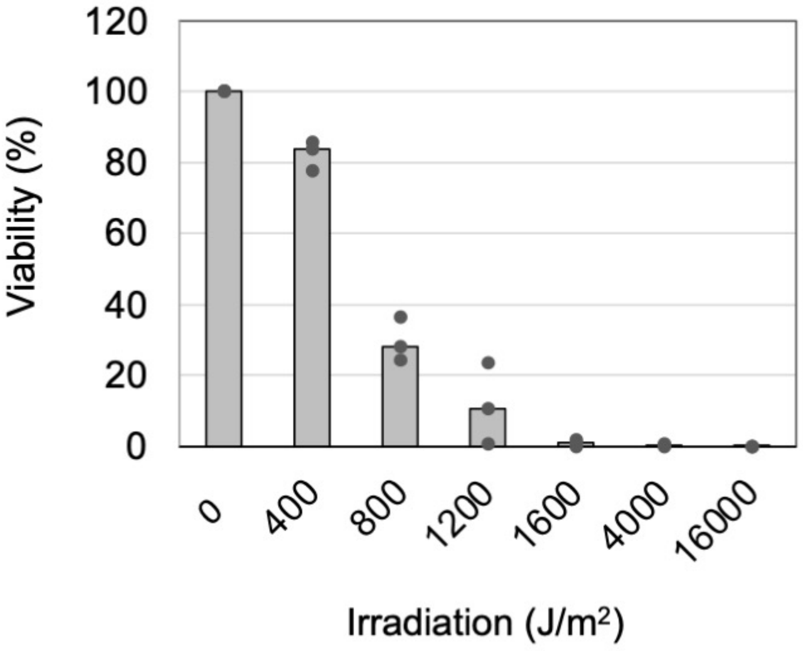

Figure S2. Survival of *Synechocystis* following UV-C irradiation. One hundred milliliters of exponentially growing cells (O.D.$_{750}$ = 0.8) were transferred to a petri dish, and the cells were irradiated with UV-C (254 nm) at a dose of 200 μW/cm^2, leading to the indicated amounts of total irradiation. Cells were harvested at various times and tested in the viability assay. The cells were spread on solid medium before and after UV-C irradiation. Surviving colonies were counted after 7 days. The data shown are the mean ± SD of values obtained in triplicate experiments.

Supplementary Figure S3

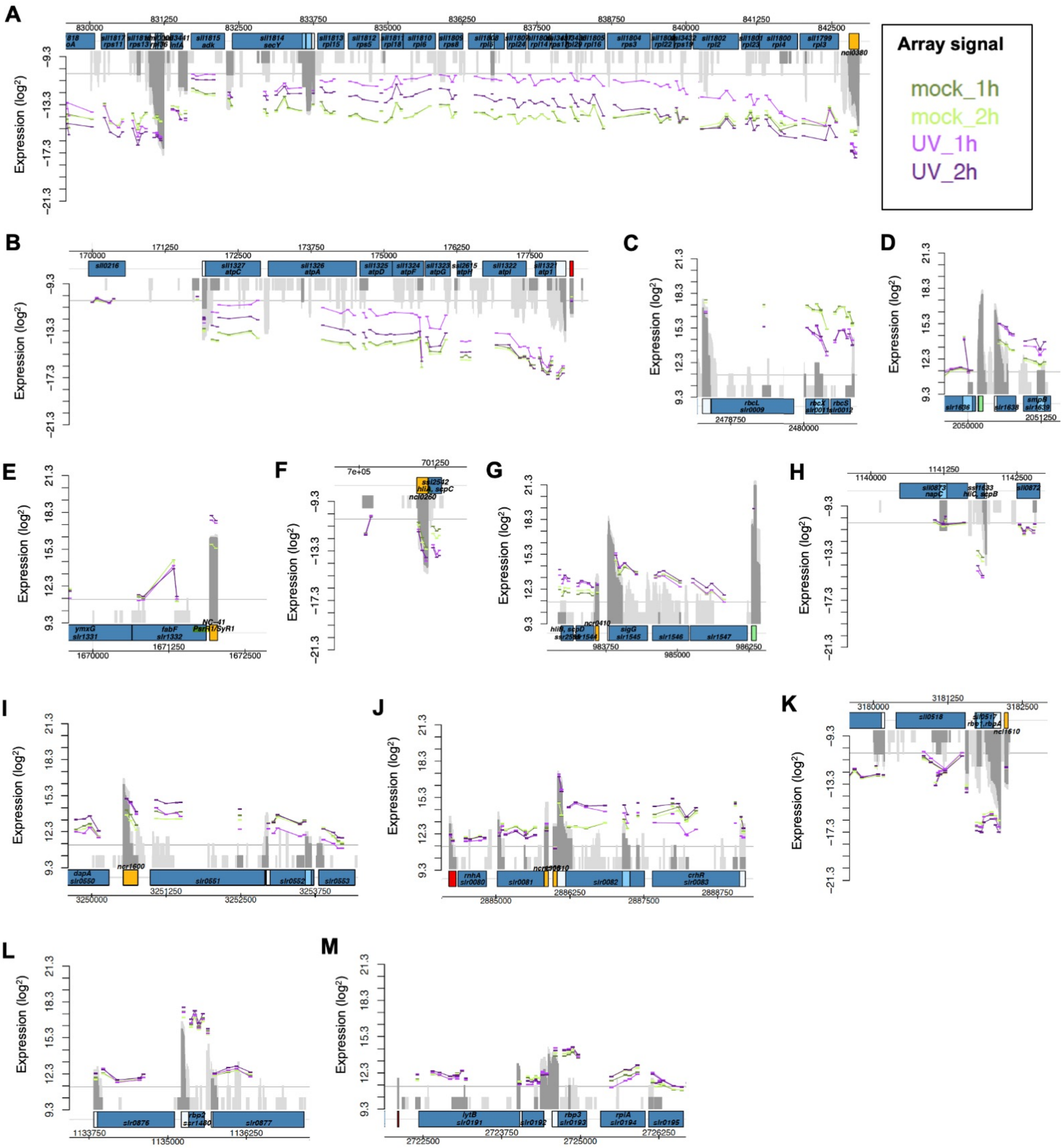

Figure S3. UV stress response in *Synechocystis* 6803. Genomic loci of significantly up- or downregulated genes were selected and are shown with the respective mapped array probes (horizontal tabs). All data are available in Supplementary Data 2. The signal intensities are given as log_2 values. (A) Operon of genes encoding ribosomal proteins (*sll1799-sll1817*) and *ncl0380*; (B) *atp* operon (*sll1321-sll1327*); (C) *rbc* operon (*slr0009-slr0012*); (D) *smpB* (*slr1639*); (E) *psrR1*; (F) *hliA* (*ssl2542*); (G) *hliB* (*ssr2595*); (H) *hliC* (*ssl1633*); (I) *rnj* (*slr0551*); (J) *rimO* (*slr0082*)-*crhR* operon; (K) *rbp1* (*sll0517*); (L) *rbp2* (*ssr1480*); and (M) *rbp3* (*slr0193*).

Supplementary Figure S4

A

-10

AAGGCTACCATTTCAGGT**TATATT**CAGTTT**A**AATTCCTTGGGTATTAGGTTTATTTTTTGACAATGCCCGGGATATTTTGGGGGGACTCCAACC

3'-RACE primer (Figs. 5 and S7)

CCTCAGGCTTGACGTCCAATGGAATCATTGACGTTGATCCAGGGATAACCAGCAAAAGTTATGCCCCTGTAGTGAATCGCCGTAAGCAGCCCCC

5'-UTR probe (Northern analysis, Fig.1)

GATTCTTCTTTTCTAATCGTTACCGGTTGTCGTTCTTGCGGTGAAGAAAAACAGATAGTTTTTTCCGTCTTAAACTGCCTCCAATACTCTTGCC

TATCTATCTCCCCCGTCCGTGCGATGGGCGGCTGTCCCCTGGGAGTTTCCCTGGGAATATCTACGGCTTTCTCCAGCAAGCTGACTGTAACCCC

TGTCCCCCAAAGGTCCCTGTTCACCCCCAATCGGGCAGCTGGGGTGGCAAAAAACGGTCACAACTATAGCAACTGAGATGACGGGAATAGTTCA

CTGGGCCAGAGGGTAGGTTGACCCTAGAGAGGAAAATATTCAGTTTACCCAATGTCCAACATTACAACATTTTTCTGTAGGCCACCTTGCCCCT

Met

GTCCACACCACTGGGACCGCTTTGGGATGGCCTTTTGAGGAATATATTT**ATGCCAAAACAAATTGTCATTGCTGAAAAACATCAGGTTGCTGCT**

GTTTTTTGGAAGGATCAAATTCAAGAATTGGTGGTGTCCACGGGCAGCCAACAGGTGGGAGATATTTATTTAGGCTTAGTTGACAATATTCTGC

ORF probe (Northern analysis, Fig. 1)

CCAGCATTGACGCCGCTTTTATTAACATTGGGGACACCGAAAAAAATGGCTTTATCCACGTCAGTGACCTCGGCCCAGTTAGATTAAGGCGCAC

CGCCGGTTCCATTTCTGAACTTTTATCTCCTCAACAAAGAGTGTTGGTGCAGGTGATGAAGGAACCCACCGGCAATAAGGGCCCCCGCCTAACT

GGAAATATCAGTATGCCGGGGCGTTATATGGTGTTGATGCCCTATGGCCGGGGAGTGAATCTTTCCCGTCGGATTAACCGGGAGGAGGAACGCA

GTCGTTTGAGGGCTTTGGCTGTGTTAATCAAGCCACCGGGCATGGGACTGCTAGTGCGGACTGAAGCGGAAGATGTGCCGGAAGATGCGATTAT

Supplementary Figure S4

B

-10

AAGGCTACCATTTCAGGT**TATATT**CAGTTT**A**AATTCCTTGGGTATTAGGTTTATTTTTTGACAATGCCCGGGATATTTTGGGGGGACTCCAACC

CCTCAGGCTTGACGTCCAATGGAATCATTGACGTTGATCCAGGGATAAC**CAGCAAAAGTTATGCCCCTGTAG**TGAATCGCCGTAAGCAGCCCCC

Primers for 5'-UTR-1 (RT-qPCR, Fig. 4)

GATTCTTCTTTTCTAATCGTTACCGGTTGTCGTT**CTTGCGGTGAAGAAAAACAGATAGTTTTT**TCCGTCTTAAAC**TGCCTCCAATACTCTTGCC**

Primers for 5'-UTR-2 (RT-qPCR, Fig. 4)

TATCTATCTCCCCCGTCCGTGCGATGGGCGGCTGTCCCCTGGGAGTTTCCCTGGGAATATCTACGGCTTTCTCCAGCAAG**CTGACTGTAACCCC**

TGTCCCCCAAAGGTCCCTGTTCACCCCCAATCGGGCAGCTGGGGTGGCAAAAAACGGTCACAACTATAGCAACTGAGATGACGGGAATAGTTCA

CTGGGCCAGAGGGTAGGTTGACCCTAGAGAGGAAAATATTCAGTTTACCCAATGTCCAACATTACAACATTTTTCTGTAGGCCACCTTGCCCCT

Met

GTCCACACCACTGGGACCGCTTTGGGATGGCCTTTTGAGGAATATATTT**ATGCCAAAACAAATTGTCATTGCTGAAAAACATCAGGTTGCTGCT**

GTTTTTTGGAAGGATCAAATTCAAGAATTGGTGGTGTCCACGGGCAGCCAACAGGTGGGAGATATTTATTTAGGCTTAGTTGACAATATTCTGC

CCAGCATTGACGCCGCTTTTATTAACATTGGGGACACCGAAAAAAATGGCTTTATCCACGTCAGTGACCTCGGCCCAGTTAGATTAAGGCGCAC

C

GGACAGCCTTGTCCTGAGTGTGGTGGTTTAGGACATTTGGTGGAACTCCCTGGCGAGAAGGGTTTTGTTTCCCTGTCCCCCACAGCGGTCAACA

Primers for ORF-1 (RT-qPCR, Fig. 4)

GTAGCATTCCCCCCCGGTTGGTGGAAAAACCGATTCTCTCTCCCCCGTTGCCAAGGTCAATGACCTGCCCAAGAAGGAAGAAGCAAAAATATC

TAGCCCCCTGGACTTACTTTTCCATCCTAATTATCAAGAGCAGGGCGATCGGGATAGTAACCGTCGTCGTCGTCGCCGTCGAGGCTCGGAGTTT

TCTGAAAAGGAAAATATTAAATCTGTGGGAATTTCCCGTAGTAAGGGTCCCAGCCCCAGCCCCACTAAGGAGAAAGTGACGGGCACTGCTCCTC

Primers for ORF-2 (RT-qPCR, Fig. 4)

CCCGCCGTGAACGGCCTTCCCGTCGAGTGGAAAAAACCTTGGTTCCGGTCGATGTCGCCATGACAACGTTGGAGCAGGATATTTATGCTCGTAT

GGGAATTTCTCCCCTAATCAAAACGGAGTACGCTGATCAAGACCCCCGCTCCTTTATGGTTTCGGTGGTTACGGCGGGAGCGGCCTTGGAGGGA

AACACTAACGGTAGTGGCAGTCTGGTTAATGCTGTTATTACAACGGTGGACAATGGAGACAACGGGGATAACGTCCCCAGTGATGGGTTGACAA

TTGTATCTGAGGTCACAGCCCCAACCCCGGTTATTGAGCAACCAAGGGAGGAAACGGTTGAGCCGGAACAGGTTGTCTTACCCCAACTTGATGA

stop

TGAAACTCCAGCCGCTCCGGTGGCAGAGGAGTCTGCTCCCATAGAAACAAAAAAACGTCCTGGCAGAAGACGGCGGCGTTCTTCAGCGGAGTAG

Supplementary Figure S4

D

-10

AAGGCTACCATTTCAGGT**TATATT**CAGTTT**A**AATTCCTTGGGTATTAGGTTTATTTTTTGACAATGCCCGGGATATTTTGGGGGACTCCAACC

Probe 1 (*in vitro* assay, Fig. 6)

CCTCAGGCTTGACGTCCAATGGAATCATTGACGTTGATCCAGGGATAACCAGCAAAAGTTATGCCCCTGTAGTGAATCGCCGTAAGCAGCCCCC

Probe 2 (*in vitro* assay, Fig. 6)

GATTCTTCTTTTCTAATCGTTACCGGTTGTCGTTCTTGCGGTGAAGAAAAACAGATAGTTTTTTCCGTCTTAAACTGCCTCCAATACTCTTGCC

TATCTATCTCCCCCGTCCGTGCGATGGGCGGCTGTCCCCTGGGAGTTTCCCTGGGAATATCTACGGCTTTCTCCAGCAAGCTGACTGTAACCCC

TGTCCCCCAAAGGTCCCTGTTCACCCCCAATCGGGCAGCTGGGGTGGCAAAAAACGGTCACAACTATAGCAACTGAGATGACGGGAATAGTTCA

CTGGGCCAGAGGGTAGGTTGACCCTAGAGAGGAAAATATTCAGTTTACCCAATGTCCAACATTACAACATTTTTCTGTAGGCCACCTTGCCCCT

Probe 3 (*in vitro* assay, Fig. 6)

Met

GTCCACACCACTGGGACCGCTTTGGGATGGCCTTTTGAGGAATATATTT**ATGCCAAAACAAATTGTCATTGCTGAAAAACATCAGGTTGCTGCT**

Probe 4 (*in vitro* assay, Fig. 6)

GTTTTTTGGAAGGATCAAATTCAAGAATTGGTGGTGTCCACGGCAGCCAACAGGTGGGAGATATTTATTTAGGCTTAGTTGACAATATTCTGC

Figure S4. Locations of the probes and primers used in this study. (A) The positions of the probes (5′ UTR and ORF, underlined in blue) used in the northern analysis (Figure 1C) and the primer used in 3′ RACE (underlined in red, Figure 5 and Figure S7) within the sequence of the *rne* (*slr1129*) ORF and its upstream region are shown. The promoter (-10), TSS, and ORF region (1-515 bp) of *rne* are shown in bold type. (B, C) The positions of the primers used in RT–qPCR (Figure 4) are shown as green arrows along the sequence of the *rne* upstream region (B) and its ORF (C). (D) The positions of probes used in the *in vitro* cleavage assay (probes 1-4, Figure 6) are underlined in red.

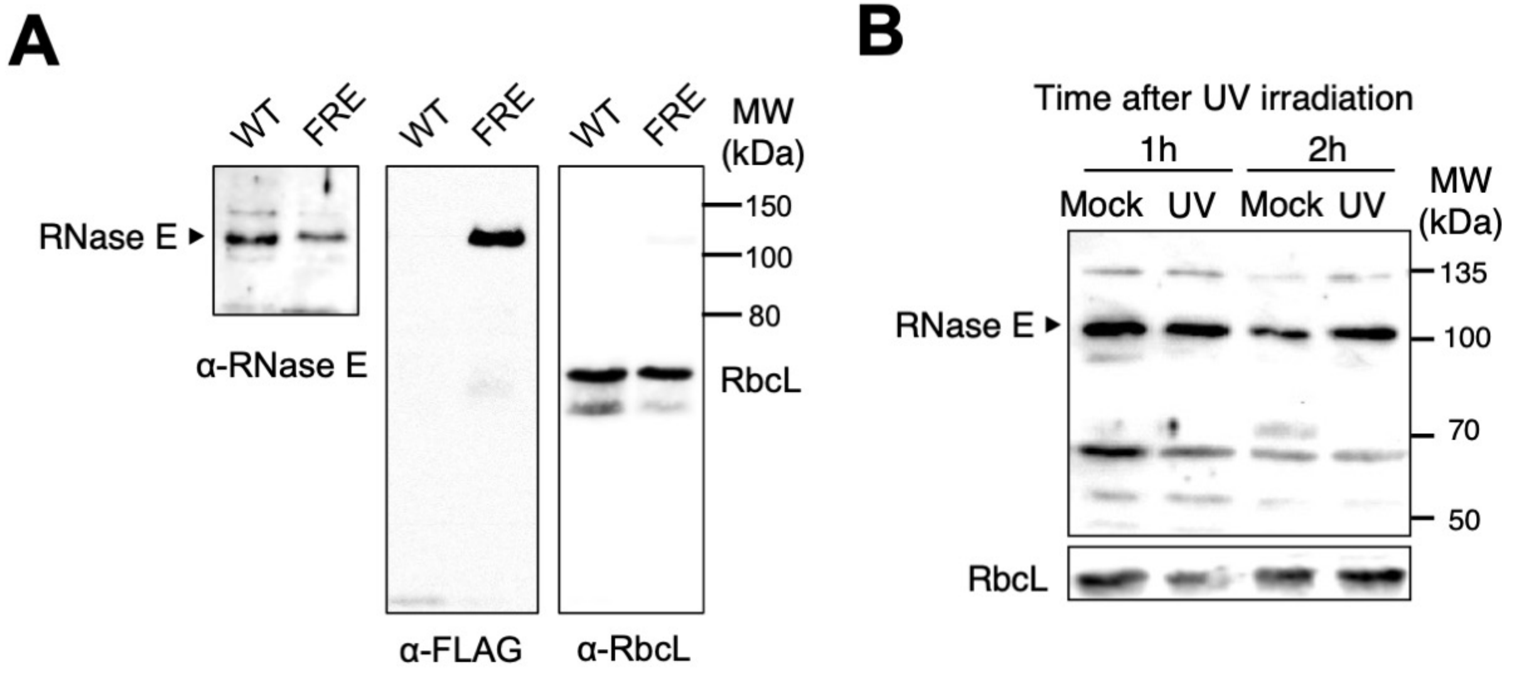

Figure S5. Analysis of RNase E protein in *Synechocystis* 6803. (A) Size estimation of RNase E. Twenty micrograms of total protein were prepared from wild-type (WT) and the engineered FRE strain expressing N-terminally FLAG-tagged RNase E from a chromosomal gene. After SDS-PAA gel electrophoresis, western blots were performed using antiseara against RNase E, FLAG tag, and RbcL, used as an internal control. To exclude nonspecific signals appearing by RNase E antibody-treatment, the upper part of the membrane (>80 kDa) was cut off and used for the western analysis. (B) Comparison of RNase E expression levels. Twenty micrograms of total protein were prepared from cultures one and two hours after UV-C treatment were subjected to western blot analysis using antibodies against RNase E (arrowhead) or RbcL.

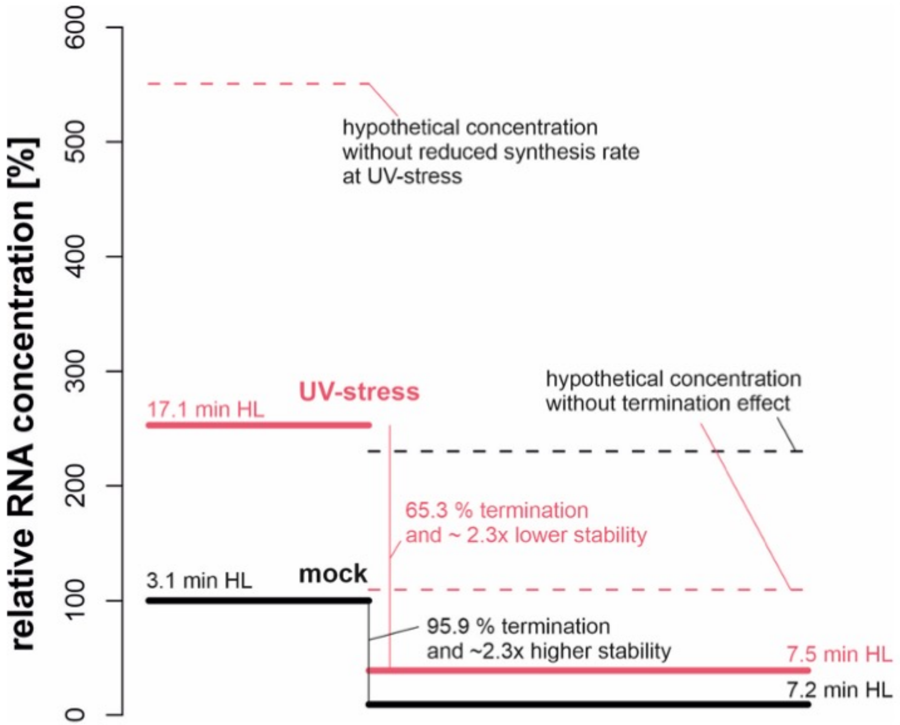

Figure S6. Schematic representation of the *rne* 5′ UTR and coding region transcript concentrations under mock and UV stress conditions, together with their respective half-lives (HL). The concentration of the 5′ UTR under mock conditions was set to 100%. The broken line in the 5′ UTR indicates the hypothetical RNA concentration under UV stress conditions if the stability had increased and the synthesis rate had remained unchanged. The broken lines in the coding region indicate the hypothetical transcript concentrations based on differences in stability without premature termination.

Supplementary Figure S7

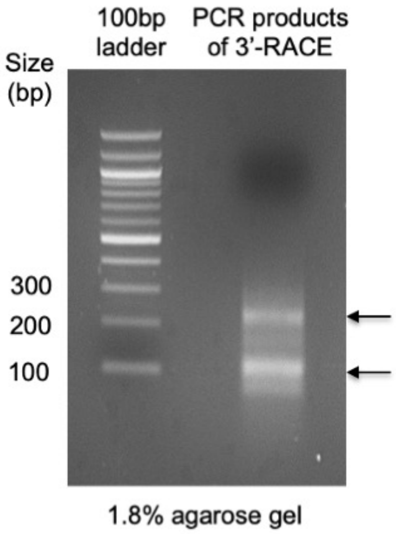

Figure S7. Electrophoresis of the PCR products used in the 3′ RACE assay. RNA samples prepared from *Synechocystis* cultures were ligated to 3′-adaptor RNAs and reverse-transcribed using a primer specific for the 3′ adaptor. Using the resulting cDNA products as a template, DNA fragments were PCR-amplified with the primer set slr1129-5UTR-f and 3RACE_Tm55 (Figure S4A and Table S1) and separated by agarose gel electrophoresis.

Supplementary Figure S8

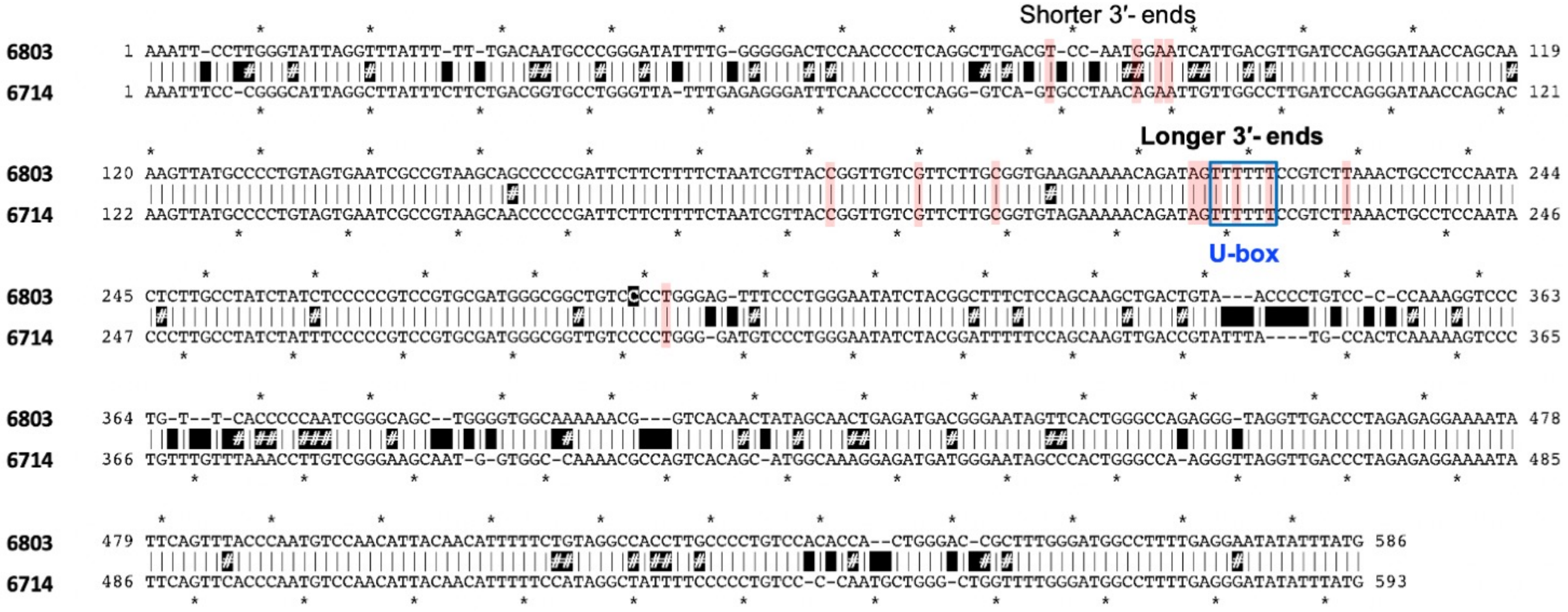

Figure S8. Alignment of *rne* 5′ UTRs in *Synechocystis* 6803 (6803) with those in *Synechocystis* 6714 (6714). The 3′ ends of the 5′ UTR transcripts identified by 3′ RACE are indicated by the red boxes. The U-rich region is boxed in blue. Mismatches between the two sequences are indicated by black boxes with hashtags.

Supplementary Tables

Table S1. Intensities and stabilities of the 5′ UTR and ORF in the *rne* transcript based on microarray signals and half-lives measured by RT–qPCR. The intensity data are based on two biological replicates. Half-life and decay were calculated based on two separately probed regions in the 5′ UTR and ORF, each with three biological replicates for each region. For the intensity the standard deviation is indicated and for half-life and decay constant the 95% confidence interval is given.

	Mock		UV 2h	
	5′UTR	ORF	5′UTR	ORF
Intensity (microarray) [AU]	$5*10^4 \pm 2.1*10^3$	$4.5*10^3 \pm 6.1*10^1$	$1.9*10^4 \pm 3*10^2$	$1.3*10^5 \pm 4.7*10^2$
Half life of transcript [min]	3.1 (2.5-4.2)	7.2 (4.2 -26.2)	17.1 (12.8-25.8)	7.5 (5.2-13.6)
Decay constant [1/min]	0.22 (0.17-0.28)	0.1 (0.03-0.17)	0.04 (0.03-0.05)	0.09 (0.05-0.13)

Table S2. Oligonucleotide primers used in this study.

Primer name	Sequence (5′ to 3′)[a]
Northern blot analysis[a]	
slr1129-5UTR-f	GACAATGCCCGGGATATTTTGGGGGACTC
slr1129-5UTR-rT7	*TAATACGACTCACTATAGGGAGA*GGAGAAAGCCGTAGATATTCCC
slr1129orf-f	GGTGGGAGATATTTATTTAGGCTTAGTTG
slr1129orf-rT7	*TAATACGACTCACTATAGGGAGA*CCGACGGGAAAGATTCACTCC
RT-qPCR	
5UTR-f1	CAGCAAAAGTTATGCCCCTGTAG
5UTR-r1	AAAAACTATCTGTTTTTCTTCACCGCAAG
5UTR-f2	TGCCTCCAATACTCTTGCCTATC
5UTR-r2	GGGACAGGGGTTACAGTCAG
ORF-f1	GTTTCCCTGTCCCCCACAG
ORF-r1	TTGGCAACGGGGGGAGAGAGAA
ORF-f2	GGAGAAAGTGACGGGCACTG
ORF-r2	AATTCCCATACGAGCATAAATATCCTGC
16S-f	GTCTGTTTCTACTTGACAAAGAGTGTAAC
16S-r	GACTTGCATGTGTTAGGCATACC
3′RACE	
3′-adaptor RNA	$_{p}$AAGAUGAAUGCAACACUUCUGUACGACUAGAGCA$_{-NH}$
slr1129-5UTR-f	GACAATGCCCGGGATATTTTGGGGGACTC
3RACE_Tm55	GTGCTCTAGTCGTACAGAAGTG
In vitro cleavage assay (RNA probes) / in vitro RNase E protection assay	
Probe 1	ATAAACCTAATACCCAAGGAA
Probe 2	TTCACTACAGGGGCATAACTTT
Probe 3	TATTTTCCTCTCTAGGGTCAAC
Probe 4	ATATATTCCTCAAAAGGCCATCC
rne5UTR-T7-fw	TAATACGACTCACTATAGGGAGAAAATTCCTTGGGTATTAGGTT
rneATG-rev	CATAAATATATTCCTCAAAAGGCC
frag1-rev	CTATCTGGGGGCGGCACCGCAAGAACGAC
frag2-fw	CCGCCCCCCAGATAGTTTTTTCCGTCTTAAAC
frag3-fw	CCCCCCAGATAGGGGGGGCCGTCTTAAACTGCCTC
as-rne 210/228	AGACGGAAAAAACTATCTG
as-rne 200/234	CAGTTTAAGACGGAAAAAACTATCTGTTTTTCTTCA

[a]Additional sequences that do not correspond to the sequences of relevant genes are italicized; restriction sites are underlined.

Supplementary Data

Supplementary Data 1. Comparative microarray data for the UV stress response compared to mock conditions in *Synechocystis* 6803 cells. Transcripts are categorized into mRNAs (labeled with their respective gene IDs), antisense RNAs (labeled “as”), potentially trans-encoded sRNAs, 5′ UTRs and transcripts derived from gene-internal segments (labeled “int”). The table displays log_2 FCs in transcript abundance under the compared conditions (UV_1 h versus Mock 1 h and UV_2 h versus Mock 2 h).

Supplementary Data 2. Transcriptomic response to UV treatment. Detailed genomic view with array probes indicated by horizontal bars connected by colored lines. The signal intensities are given as log_2 values. The graphs shown in gray represent RNA sequencing data given as log_2 read numbers; these were extracted from the previous genome-wide mapping of TSSs (82).
